# Transcriptional and epigenetic characterization of a new in vitro platform to model the formation of human pharyngeal endoderm

**DOI:** 10.1186/s13059-024-03354-z

**Published:** 2024-08-08

**Authors:** Andrea Cipriano, Alessio Colantoni, Alessandro Calicchio, Jonathan Fiorentino, Danielle Gomes, Mahdi Moqri, Alexander Parker, Sajede Rasouli, Matthew Caldwell, Francesca Briganti, Maria Grazia Roncarolo, Antonio Baldini, Katja G. Weinacht, Gian Gaetano Tartaglia, Vittorio Sebastiano

**Affiliations:** 1 Department of Obstetrics & Gynecology, Stanford University, Stanford, CA 94305 USA; 2 Institute for Stem Cell Biology and Regenerative Medicine (ISCBRM), Stanford School of Medicine, Stanford, CA 94305 USA; 3 Department of Biology and Biotechnology Charles Darwin, Sapienza University of Rome, 00185 Rome, Italy; 4 Center for Life Nano- & Neuro-Science, Fondazione Istituto Italiano Di Tecnologia (IIT), 00161 Rome, Italy; 5 Biomedical Informatics Program, Department of Biomedical Data Science, Stanford University, Stanford, CA 94305 USA; 6 Department of Genetics, School of Medicine, Stanford University, Stanford, CA 94305 USA; 7 Cardiovascular Institute and Department of Medicine, Stanford University, Stanford, CA 94305 USA; 8 Division of Hematology, Oncology, Stem Cell Transplantation, and Regenerative Medicine, Department of Pediatrics, Stanford School of Medicine, Stanford, CA 94305 USA; 9 Center for Definitive and Curative Medicine (CDCM), Stanford School of Medicine, Stanford, CA USA; 10 Department of Molecular Medicine and Medical Biotech., University Federico II, 80131 Naples, Italy; 11 Center for Human Technology, Fondazione Istituto Italiano Di Tecnologia (IIT), 16152 Genoa, Italy

**Keywords:** Pharyngeal Endoderm, Retinoic Acid, Transcriptomics, Epigenomics, Transcription Factors, Human Development

## Abstract

**Background:**

The Pharyngeal Endoderm (PE) is an extremely relevant developmental tissue, serving as the progenitor for the esophagus, parathyroids, thyroids, lungs, and thymus. While several studies have highlighted the importance of PE cells, a detailed transcriptional and epigenetic characterization of this important developmental stage is still missing, especially in humans, due to technical and ethical constraints pertaining to its early formation.

**Results:**

Here we fill this knowledge gap by developing an in vitro protocol for the derivation of PE-like cells from human Embryonic Stem Cells (hESCs) and by providing an integrated multi-omics characterization. Our PE-like cells robustly express PE markers and are transcriptionally homogenous and similar to in vivo mouse PE cells. In addition, we define their epigenetic landscape and dynamic changes in response to Retinoic Acid by combining ATAC-Seq and ChIP-Seq of histone modifications. The integration of multiple high-throughput datasets leads to the identification of new putative regulatory regions and to the inference of a Retinoic Acid-centered transcription factor network orchestrating the development of PE-like cells.

**Conclusions:**

By combining hESCs differentiation with computational genomics, our work reveals the epigenetic dynamics that occur during human PE differentiation, providing a solid resource and foundation for research focused on the development of PE derivatives and the modeling of their developmental defects in genetic syndromes.

**Supplementary Information:**

The online version contains supplementary material available at 10.1186/s13059-024-03354-z.

## Background

Human embryogenesis is characterized by the progression of highly dynamic and temporal stages involving sequential chromatin and transcriptional changes, driven by extracellular and intracellular signaling pathways that occur in a cell type- and stage-dependent manner [1–3]. The proper regulation of these processes is essential for the accurate, robust, and reproducible development of progenitor-like cells into distinct cell types, forming a cooperative and cohesive network of physiological systems [4]. Studying these processes is crucial since they provide insights that can be leveraged to identify key signaling pathways that coordinate human development, and to understand how their disruption contributes to developmental and congenital diseases [2, 5–7]. Human Embryonic Stem Cells (hESCs) have greatly improved the ability to study human development and developmental-related diseases, thanks to their capability to self-renew and differentiate into all cell types of the human body [4, 8, 9] and the ease of derivation from genetically mutated somatic cells. However, to harness the full potentiality of this platform, it is essential to mimic the signals that occur in vivo during hESCs differentiation to direct the development of these cells to specific lineages.

One such lineage is the Pharyngeal Endoderm (PE), which contributes to the Pharyngeal Apparatus (PA) in vertebrates. This structure is highly conserved among vertebrates, and it is formed between E8.5–10.5 in mice and E21-28 in humans, with the contribution of cells from all three germ layers [10–12]. The PE, which originates from the anterior-most region of the foregut, is considered the main driver orchestrating the development of the PA. This is primarily due to the formation of the Pharyngeal Pouches (PPs), valley-like structures within the PA, which emerge thanks to the out-pocketing of the PE [11, 13–15]. The PPs serve as a microenvironment for physiological development and are essential for the morpho-patterning of important organs and structures such as the lining of the pharynx, palatine tonsils, inner ear, parathyroids, thyroid glands, ultimobranchial bodies, and the thymus [5]. Impairment of PE formation during PA development was found to be the cause of severe developmental-related abnormalities that are responsible for one-third of all congenital disorders, mainly being tied to a weakened or absent formation of this microenvironment [16]. Among them, 22q11.2 Deletion Syndrome (22q11.2DS), the most common microdeletion syndrome, which affects 1/2–4000 live births [6, 7, 17], has been linked to defective PE development. Despite the fundamental role of the PE during PA development and its connection with developmental diseases, the transcriptional and epigenetic dynamics which characterize this cell type remain poorly studied. To derive functional PE cells, the in vitro differentiation protocols should mimic the sequential origin of intermediate cell types occurring during in vivo development [5]. These stages include the specification into Definitive Endoderm (DE), the patterning into Anterior Foregut Endoderm (AFE), and the subsequent specification into PE. Although many groups have worked on the generation of DE, AFE, and PE lineages [18–23], most of the protocols available so far were able to generate cells that displayed only a moderate level of expression of a handful of PE markers (PAX9, SOX2, FOXA2, TBX1) [18, 19] and, in some cases, the cells expressed markers of the DE stage that should have been instead silenced at the PE stage (i.e. SOX17) [23]. Even more importantly, none of these works have extensively characterized the transcriptome and the epigenome of the PE cell stage, since the PE stage was used only as an intermediate substrate to obtain more differentiated cell types such as thymic or parathyroid cells [21, 22, 24], and sometimes bypassed [25], leaving a gap of critical information necessary to study this process.

Retinoic Acid (RA) signaling was shown to be involved in the regulation of pharyngeal patterning [26] and in the proper formation of the third and fourth pharyngeal arches [27, 28]. Furthermore, alterations in RA concentration cause defects in the development of the thymus and parathyroids, both structures originating from the 3rd pharyngeal pouches [29–31] and complete loss of RA synthesis in the developing embryo recapitulates most of the phenotype of the 22q11.2DS [31]. RA has been implemented in several differentiation protocols to ultimately derive later-stage thymus cells [21, 22, 24] but, again, an in-depth molecular and epigenetic characterization of RA role is still missing. Under the hypothesis that RA plays a crucial and yet under-investigated role in the development of PE cells in vitro, we developed and validated a defined monolayer differentiation protocol using small molecules in combination with a specific RA concentration and chemically defined media to generate a transcriptionally homogeneous cell population expressing all the PE markers known in literature so far. By combining downstream analysis such as bulk and single-cell RNA-Seq, the Assay for Transposase-Accessible Chromatin with Sequencing (ATAC-Seq), and Chromatin Immunoprecipitation followed by Sequencing (ChIP-Seq) of histone modifications, we were able to deeply characterize the transcriptomic and the epigenomic landscape of our PE-like cells, to generate a transcription factor network (TFN) and to identify previously unknown CIS-regulatory elements likely responsible of the proper PE differentiation. In addition, we dissected the transcriptional and epigenetic contribution of RA in PE specification, elucidating in part the role of RA in human pharyngeal development. Our data provide a detailed and rich set of information on human specific PE regulation that cannot otherwise be achieved due to the technical and ethical constraints in obtaining and studying the in vivo human PE cells. Our work offers a robust discovery platform and a valuable resource, enabling the functional characterization of previously undiscovered regulatory elements. Additionally, our in vitro PE differentiation protocol serves as a potent tool for investigating this critical, albeit largely unexplored, intermediate developmental stage.

## Results

### Differentiation of hESCs into *bona fide *PE cells by the dynamic exposure of AFE cells to RA signaling and transcriptional characterization

**Fig. 1:**
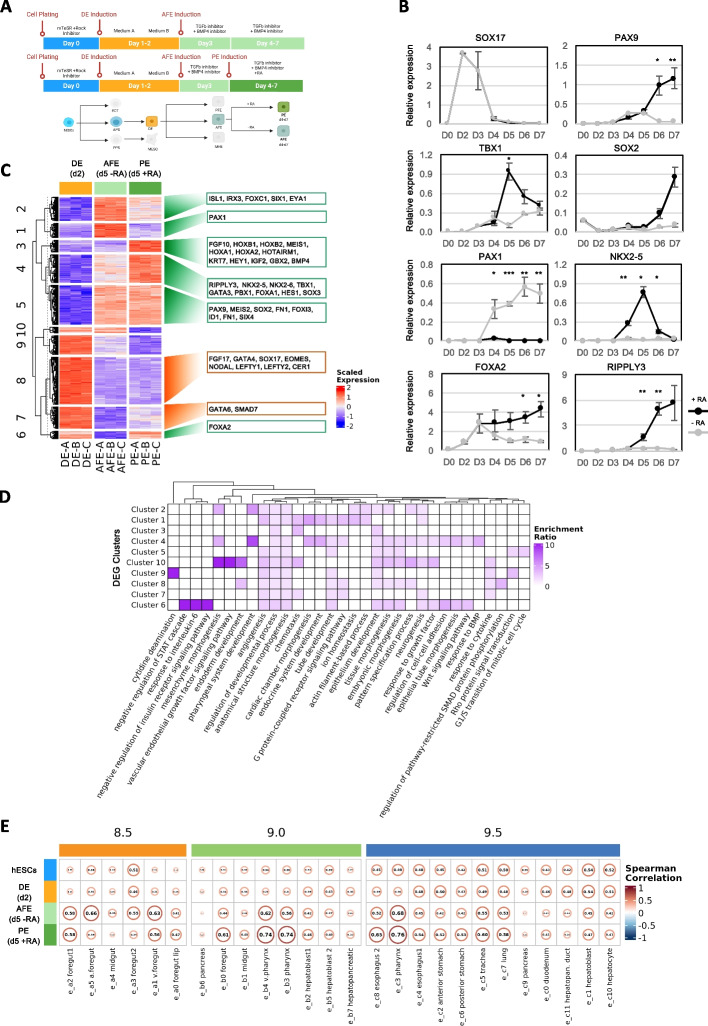
Bulk RNA-Seq analysis reveals extensive transcriptomic changes during the in vitro differentiation of PE cells. **A** Schematic representation of the protocol for the in vitro differentiation of hESCs into Pharyngeal Endoderm cells. Created with BioRender.com.** B** RT-qPCR time course analysis showing the relative expression of DE and PE specific markers in the presence (black) or absence (grey) of RA. Data were normalized on PDGB expression and represent means ± SEM of three independent time-course experiments. *p < 0.05, **p < 0.01, ***p < 0.001, unpaired Student’s t-test. **C** Heatmap showing the expression in DE (d2), AFE (d5 -RA), and PE (d5 +RA) samples of the greatly varying (strict) DEGs identified in DE vs AFE and DE vs PE contrasts, as well as their separation in ten clusters produced via k-means clustering. Hierarchical clustering of the ten clusters is also shown; see also Additional File 3. Known markers belonging to each cluster are shown in the boxes on the right. The expression values reported in the heatmap correspond to row-scaled (Z-score) rlog-transformed count data. **D** Heatmap showing the results of the GO BP term enrichment analysis performed on the ten gene clusters shown in C; the color intensity in each cell is proportional to the Enrichment Ratio. The heatmap reports only a set of the significantly enriched categories (FDR < 0.05 in at least one cluster), selected in order to reduce redundancy; Enrichment Ratio is plotted only when FDR < 0.25; see also Additional File 4. **E** Spearman correlation matrices showing the similarity between hESCs, DE, AFE, and PE cells (rows) and embryonic mouse foregut endodermal cell type clusters identified by Han and colleagues (columns), based on the expression of the top 10 transcription factors enriched in each cluster. Text and circle width in each cell of the matrices are proportional to the absolute value of the Spearman correlation. Each matrix corresponds to a different murine developmental stage (day 8.5, day 9.0, and day 9.5)

In order to generate a robust and homogenous population of PE-like cells, we attempted to build upon our previously published protocol for the generation of functional AFE cells (20). H9 hESCs (d0) were differentiated into DE (d2) by using the PSC Definitive Endoderm Induction kit, formulated by using the findings from our previous work (20), which is now routinely used for the generation of highly pure DE cells. After 24 h in Medium A and 24 h in Medium B (Fig. 1A), the DE (d2) cells were anteriorly patterned into AFE by dual inhibition of TGFb and BMP4 for 24 h generating AFE (d3) cells [20]. Given the previously described contribution of RA in the PE formation, we tested the hypothesis that the addition of RA was necessary and sufficient to activate a gene regulatory network able to induce the differentiation of AFE into PE-like cells. To test our hypothesis, AFE cells were cultured with increasing concentrations of RA (0–800 nM) and checked for the expression of the known PE markers TBX1, NKX2-5, PAX9, PAX1, and RIPPLY3 after 48 h of exposure (d5) (Additional File 1: Fig. S1A). The titration showed that 50–200 nM (see Methods section for details) represents the optimal concentration, leading to a proper combination of expression of the tested PE markers (Additional File 1: Fig. S1A). With 50–200 nM as the ideal concentration of RA, we then sought to identify the optimal exposure time of cells to RA by evaluating the expression of such markers during a 7 days long time course of differentiation and in the presence or in the absence of RA, which was added for 24, 48, 72, or 96 h (Fig. 1A). This analysis led us to choose 48 h as the optimal window of exposure to RA for obtaining PE cells, based on the expression peak of several PE markers (Fig. 1B). Notably, SOX17, a specific marker of the DE stage, was properly downregulated during the differentiation in both conditions. Of note, in the presence of RA PAX1 was still expressed but downregulated compared to the RA-treated condition, in line with the dynamic expression observed in vivo [29]. To gain a more unbiased and comprehensive understanding of the transcriptional changes in response to RA, we decided to deeply characterize and compare the entire transcriptome of our cells. To do this, polyadenylated RNA from hESCs (d0), DE (d2), AFE (d5 -RA), and PE (d5 +RA) cells was collected and submitted to a bulk RNA-Seq analysis. As shown by the sample clustering analyses (Additional File 1: Fig. S1B and C), d5-AFE (d5 -RA) and d5-PE (d5 +RA) samples have similar but distinct gene expression profiles, significantly different from those of hESCs and DE samples, which form two separate clusters. Differential gene expression analysis between each pair of conditions allowed us to identify 7226 differentially expressed genes (DEGs) (Additional File 1: Fig. S1D and Additional File 2: Table S1). Given our interest in identifying the transcriptomic changed induced by the addition of RA, we focused on the DEGs with great variation in expression (see Methods) among the DE (d2), PE (d5 +RA), and AFE (d5 -RA) conditions, for a total of 4578 genes. Such genes were grouped into ten clusters based on their expression trends (Fig. 1C and Additional File 3: Table S2). GO term [32] enrichment analysis was performed on each cluster to gain insight into the function of each class of DEGs **(**Fig. 1D and Additional File 4: Table S3). While we found that genes from all the clusters were involved in Biological Process (BP) categories related to development and morphogenesis, we also observed that the “pharyngeal system development” function was highly enriched in cluster 4, which is composed of genes whose expression is induced from DE (d2) to AFE-PE (d5) and particularly boosted by the presence of RA. In agreement with this functional enrichment analysis, all the known marker genes of the PE are upregulated during the transition from DE (d2) to AFE (d5 -RA) and PE (d5 +RA), and most of them are induced by the presence of RA **(**Fig. 1C, green boxes, and Additional File 3: Table S2) [5, 20, 21, 33–53], while DE-specific markers are properly downregulated (Fig. 1C, orange boxes, and Additional File 3: Table S2) [54–60]. To further confirm the reliability of our protocol in activating a PE-specific transcriptional network, we identified the functional categories enriched among upregulated and downregulated genes in DE (d2) vs AFE (d5 -RA) and DE (d2) vs PE (d5 +RA) contrasts via GSEA [61]. As expected, for both comparisons, we found the “endoderm development” GO Biological Process (BP) category to be enriched among the downregulated genes (i.e. genes more expressed in DE) as well as the “pharyngeal system development” and related categories to be over-represented among the upregulated genes (Additional File 1: Fig. S1E, top panel). Furthermore, by performing GSEA on the AFE (d5 -RA) vs PE (d5 +RA) contrast to highlight the major transcriptional changes induced by the addition of RA, we found the “activation of HOX genes during differentiation” and “activation of anterior HOX genes” Reactome pathways [62] as the most enriched among the upregulated genes (Additional File 1: Fig. S1E, bottom panel). Finally, we compared the expression profile of our cells with that of the mouse in vivo counterparts at embryonic days 8.5, 9.0, and 9.5 by taking advantage of the single-cell transcriptomic data previously published by Han and colleagues [34], who generated a spatiotemporal map of endoderm and mesoderm development during murine foregut organogenesis (Additional File 1: Fig. S1F). Interestingly, we found HOXA1, HOXA2, HOXB1, and HOXB2 genes, the most definitive regulators of Anterior–Posterior patterning, to be specifically upregulated in our PE cells and in the Pharyngeal endoderm clusters of mouse in vivo development (clusters e_b3 and e_c3) (Additional File 1: Fig. S1G). We then compared our cells with the different endodermal clusters identified by Han and colleagues (Fig. 1E) by looking at the transcription factor (TF) expression profile. Notably, we found AFE (d5 -RA) cells to be like the corresponding anterior foregut cluster in mouse **(**Fig. 1E, cluster e_a5), while our PE (d5 +RA) cells showed high similarity with the Pharyngeal Endoderm clusters at day 9.0 (Fig. 1E, clusters e_b3 and e_b4) and at day 9.5 (Fig. 1E, cluster e_c3). Interestingly, by looking at the functional enrichment of the e_b3, e_b4, and e_c3 gene markers which are also abundantly expressed (> 5 TPM) in our PE (d5 +RA) cells, we found “pharyngeal system development” to be the most enriched category, followed by other development-related categories (Additional File 1: Fig. S1H and Additional File 4: Table S3), further confirming that, based on their gene expression profile, these cells can be considered *bona fide* human Pharyngeal Endodermal cells.

To rule out the possibility that our PE cells could be mapped to a stage of murine development later than E9.5, we compared them with the endodermal cell clusters obtained by Magaletta and colleagues [63] through scRNA-Seq analysis performed on an endoderm developmental time course ranging from day 9.5 to day 12.5. By assessing the similarity using the top 10 TF markers expressed in each cluster, we confirmed that our PE cells show the maximum concordance with immature pharynx cells at E9.5 (Additional File 1: Fig. S1I).

### scRNA-Seq analysis reveals a homogeneous transcriptional signature for PE (d5 +RA) cells which is distinct from the AFE (d5 -RA) condition

**Fig. 2:**
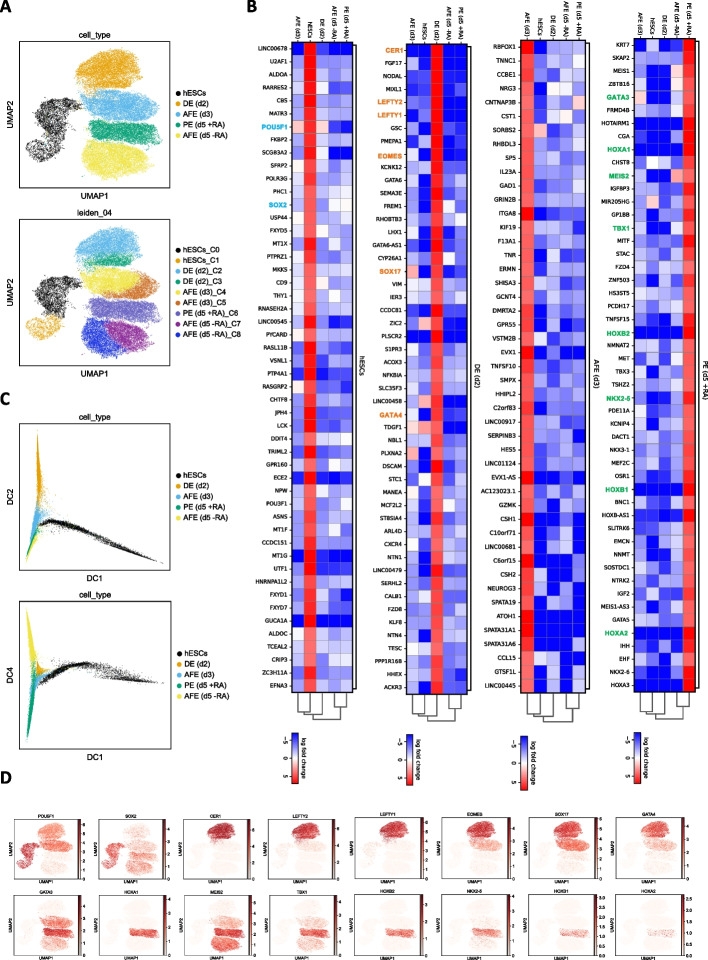
Single-cell RNA-Seq analysis demonstrates the transcriptional homogeneity of the in vitro-derived PE (d5 +RA) cells. **A** UMAP-based visualization of scRNA-Seq data produced from hESCs, DE (d2), AFE (d3), AFE (d5 -RA), and PE (d5 +RA) cells, with cells colored based on the differentiation stage (top panel) and Leiden clustering (with resolution 0.4) (bottom panel). **B** Matrix plots showing the log2(FC) of the top 50 DEGs identified in the hESCs, DE (d2), AFE (d3), AFE (d5 -RA), and PE (d5 +RA) clusters (log2(FC) ≥ 4). Known markers for the hESCs, DE, and PE stages are highlighted in blue, orange and green, respectively. **C** Diffusion map of scRNA-Seq data colored according to the differentiation stage. We show the second and the fourth diffusion components (DC) versus the first in the top and bottom panels, respectively. **D** UMAP plot representation of the single-cell gene expression (log[norm.counts + 1]) of the marker genes highlighted in B, in hESCs, DE (d2), AFE (d3), AFE (d5 -RA), and PE (d5 +RA) cells

To assess the transcriptionally homogeneity of our cell population and confirm the role of the RA in driving the transition into PE at the single-cell level, we performed single-cell transcriptomic profiling on hESC (d0), DE (d2), AFE (d3), AFE (d5, -RA), and PE (d5, +RA) (see Methods for further details). Leveraging the expression patterns of highly variable genes, we utilized the Uniform Manifold Approximation and Projection (UMAP) method [64] for visualization in two dimensions. In alignment with our findings from bulk RNA-Seq data, this analysis clearly displayed a distinct separation of cells based on their developmental stages (Fig. 2A, top panel, and Additional File 5: Fig. S2A), emphasizing the contrast between AFE (d5 -RA) and PE (d5 +RA).

We then explored subtle transcriptional heterogeneity using the Leiden clustering algorithm [65], which identified nine distinct clusters based on transcriptomic similarities (Fig. 2A, bottom panel). Interestingly, the PE cells unified into a single cluster, marking them as the final cell type to segregate within the hierarchy (Additional File 5: Fig. S2B). This observation supports our hypothesis that our in vitro differentiation protocol leads to the formation of a transcriptionally homogenous cell population. Notably, by comparing the DEGs between the PE1 and PE2 subclusters generated when increasing the resolution of the Leiden clustering, we did not identify any genes associated with PE regulation; instead, the DEGs were predominantly related to cell cycle processes (Additional File 5: Fig. S2C).

The top 50 DEGs identified at each stage confirmed the stage-specific upregulation of known hESCs, DE, and PE markers (Fig. 2B) corroborating the bulk RNA-Seq results.

A diffusion map computed from scRNA-Seq data showed that cells align along a continuous trajectory (Fig. 2C), with the first two diffusion components (DC1 and DC2), shown at the top, dominated by the hESCs heterogeneity and the difference between the DE (d2) and the cell types emerging in the next days of differentiation, respectively. Interestingly, the fourth diffusion component, shown at the bottom, clearly highlights the presence of a branching point that separates the AFE (d5 -RA) from the PE (d5 +RA).

Finally, we selected some of the known hESCs, DE, and PE marker genes that emerged from the analysis shown in Fig. 2B, and we reported their expression in UMAP plots, which show that their expression is distributed across cells of the expected cell type, i.e. they are not differentially expressed between the sub-clusters of a cell type. Despite the uniform distribution, the expression of some PE markers was not detected across all cells (this is expected due to the known limitations of the scRNA-Seq, which typically captures only 20–30% of the most expressed genes). To confirm the uniform activation, we performed immunofluorescence (IF) on FOXA2, NKX2-5, GATA3, SOX2, and SOX17 markers. Additionally, we generated a TBX1-mRUBY reporter cell line to confirm the uniformity of TBX1 expression. Both IF and Flow analysis show that all our cells homogeneously express the analyzed markers (Additional File 5: Fig. S2D and E). Taken together, these findings suggest that our PE (d5 +RA) cells represent a homogenous cell population that properly differentiates into the expected cell type and that is transcriptionally distinct from the AFE (d5 -RA).

### Exposure to Retinoic Acid induces chromatin accessibility changes accompanying transcriptomic variations during PE differentiation

To provide a clear picture of the putative regulatory regions likely responsible for the activation of a PE-specific transcriptional program in response to RA signaling, we decided to deeply characterize and investigate the epigenetic landscape of DE, AFE (d5 -RA), and PE (d5 +RA) cells via ATAC-Seq. Peak calling was performed for each sample to find accessible regions; consensus peaks for each condition and peaks in common between different conditions were subsequently identified. Following this approach, we discovered 107,569 accessible regions in DE, 95,231 in AFE (d5 -RA), and 80,441 in PE (d5 +RA), 50,015 of which are in common among the three conditions (Additional File 6: Fig. S3A, top panel). Clustering analyses performed on ATAC-Seq samples clearly showed that our cells have a distinct epigenomic landscape at each stage, the AFE (d5 -RA) and PE (d5 +RA) cells being more similar to each other than to DE cells (as also observed from transcriptomics data), confirming the quality and the reproducibility of each replicate (Additional File 6: Fig. S3B and C).

**Fig. 3:**
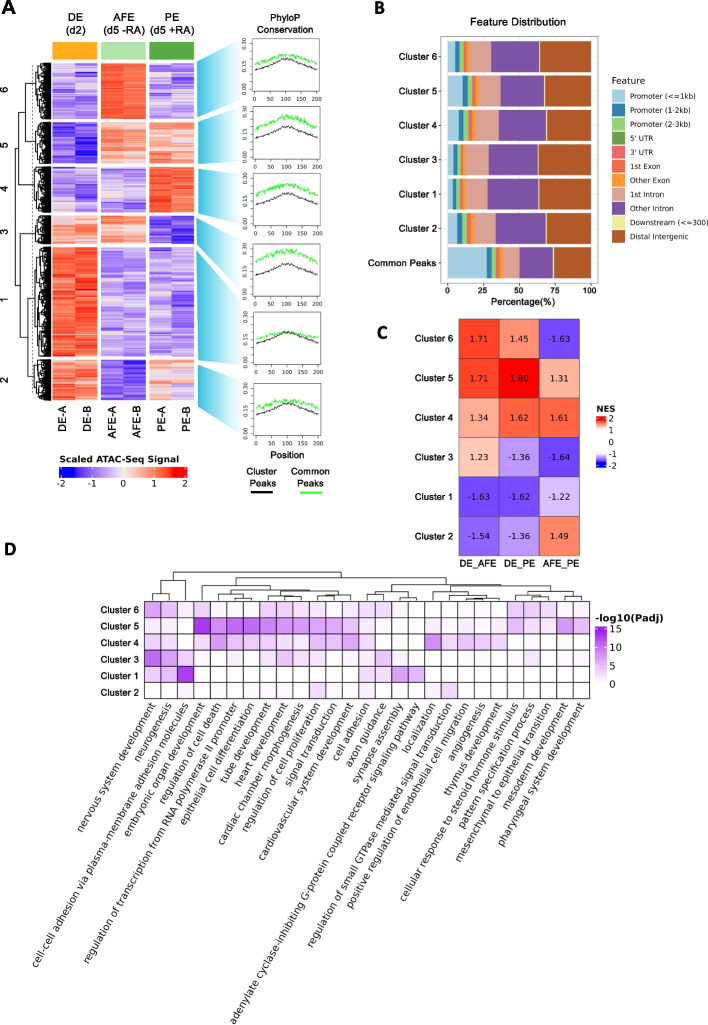
ATAC-Seq analysis reveals functional differentially accessible regions among DE, AFE, and PE cell types. **A** Heatmap showing the ATAC-Seq signal in DE, AFE, and PE samples of the DARs identified in all contrasts, as well as their separation in six clusters produced via k-means clustering. Hierarchical clustering of the six clusters is also shown. Average PhyloP conservation scores, calculated for each genomic position within DARs and Common peaks, are shown in the plots on the right. The ATAC-Seq signal values reported in the heatmap correspond to row-scaled (Z-score) log2-transformed library size-normalized count data. **B** Bar plot showing the genomic annotation of DARs belonging to each cluster shown in Fig. 3A and Common ATAC-Seq peaks. Each genomic feature is represented by a specific color shown in the legend. **C** Table showing the Normalized Enrichment Scores (NES) calculated performing GSEA on each differential gene expression contrast (DE vs AFE, DE vs PE, and AFE vs PE) and using sets of expressed protein-coding genes having a TSS in proximity (< 50 kb) of cluster-specific DARs. Positive NES: the gene set is enriched among the upregulated genes; Negative NES: the gene set is enriched among the downregulated genes. **D** Heatmap showing the results of the GREAT analysis performed on the six DAR clusters shown in A; the color intensity in each cell is proportional to the adjusted *p*-value. The heatmap reports a set of the significantly enriched GO BP terms (adjusted *p*-value < 0.01 in at least one cluster), selected in order to reduce redundancy

To locate the genomic regions responsible for each stage-specific epigenetic profile, we performed a differential accessibility analysis between each pair of conditions, which led to the identification of differentially accessible regions (DARs, see Methods). For each comparison, DARs were classified into Gain (log2[FC] > 1) and Lose (log2[FC] < -1) peaks (Additional File 6: Fig. S3A, bottom panel, and Additional File 7: Table S4). Based on read coverage, DARs were further grouped into six different clusters (Fig. 3A); each of them shows a distinct behavior during differentiation, indicating that the chromatin accessibility is actively changing during the induction of the differentiation and that is actively responding to the addition of RA. Concordantly with the number of consensus peaks observed in each condition, the largest cluster is the one composed of regions whose accessibility decreases in the transition from DE to AFE (d5 -RA) and PE (d5 +RA) (cluster 1, 30,927 peaks), indicating that a significant proportion of genomic regions are closed during differentiation. Interestingly, an analysis of the evolutionary conservation in vertebrates performed on DARs from each cluster showed that, overall, DARs tend to be more conserved than regions with no significant change in accessibility (Common peaks). Moreover, regions gaining accessibility in response to differentiation and regions specifically open or closed upon the addition of RA (clusters 3, 4, and 5) have higher levels of sequence conservation than other DARs, suggesting an evolutionarily conserved function for such sequences (Fig. 3A) and that the mechanism through which RA promotes the transcriptional and epigenetic maturation of PE could be similar in other species. Another feature that distinguishes DARs from Common peaks is their genomic distribution with respect to gene elements (Fig. 3B): while ~ 33% of Common peaks are in promoter regions, DARs from all clusters are more often located outside such regions. This is particularly evident for peaks losing accessibility in the transition from DE to PE (d5 +RA) (cluster 1 and cluster 3, ~ 9% of the peaks falling in promoter regions), and less so for DARs specifically open in both AFE (d5 -RA) and PE (d5 -RA) (cluster 5) and only in PE (d5 +RA) (cluster 4) (~ 17% and ~ 14% of the peaks falling in promoter regions, respectively). DARs not overlapping with promoter regions could regulate the expression of nearby genes by acting as enhancers. To test this hypothesis, for each DAR cluster we identified a gene set composed of genes whose transcription start sites (TSSs) are less than 50 kb away from any of the peaks of the cluster; then, for each differential gene expression contrast, we performed a GSEA to evaluate whether cluster-specific gene sets are enriched among the upregulated or the downregulated genes (Fig. 3C). This analysis showed that Gain DARs tend to be located near upregulated genes, while Lose DARs are found in the proximity of downregulated genes. Additionally, examining the ATAC-Seq peaks found in proximity of the gene markers highlighted in Fig. 1C, we confirmed that changes in gene expression are often mirrored by variations in chromatin accessibility (Additional File 6: Fig. S3D). The significance of DARs, inferred by the function of their nearby genes, was investigated by performing GREAT analysis [66] on each peak cluster (Fig. 3D). Interestingly, for clusters 5 and 4 we found a clear functional enrichment in GO BP terms related to the development of the Pharyngeal Apparatus (with cluster 5 showing enrichment for genes involved in thymus development, a downstream cell type originating from the PE), further supporting the notion that chromatin is dynamically inducing the establishment of a transcriptional program promoting the differentiation of our cells into PE progenitors. To gain insight into how changes in TSS accessibility contribute to the observed variations in gene expression, we evaluated the ATAC-Seq read coverage around the TSS of previously identified DEGs and of non-DEGs. Interestingly, we observed a general increase in the TSS accessibility in the transition from DE to AFE (d5 -RA) and PE (d5 +RA) both in DEGs and non-DEGs (Additional File 6: Fig. S3E), and some enrichment of Gain and Lose peaks in the promoters of upregulated and downregulated genes, respectively (Additional File 6: Fig. S3F). However, since most of the DEG TSSs overlap with Common ATAC-Seq peaks (Additional File 6: Fig. S3F), it appears that dynamic promoter accessibility is not a dominant effect in the regulation of gene expression and that the chromatin accessibility changes responsible for the variations in gene expression mainly occur in regions located outside of promoters. In support of this, we found several DE and PE markers whose nearby regions are respectively closing and opening during the differentiation process (Additional File 6: Fig. S3G and H).

### Stage-specific transcription factor activation correlates with chromatin changes on predicted TF binding sites

**Fig. 4:**
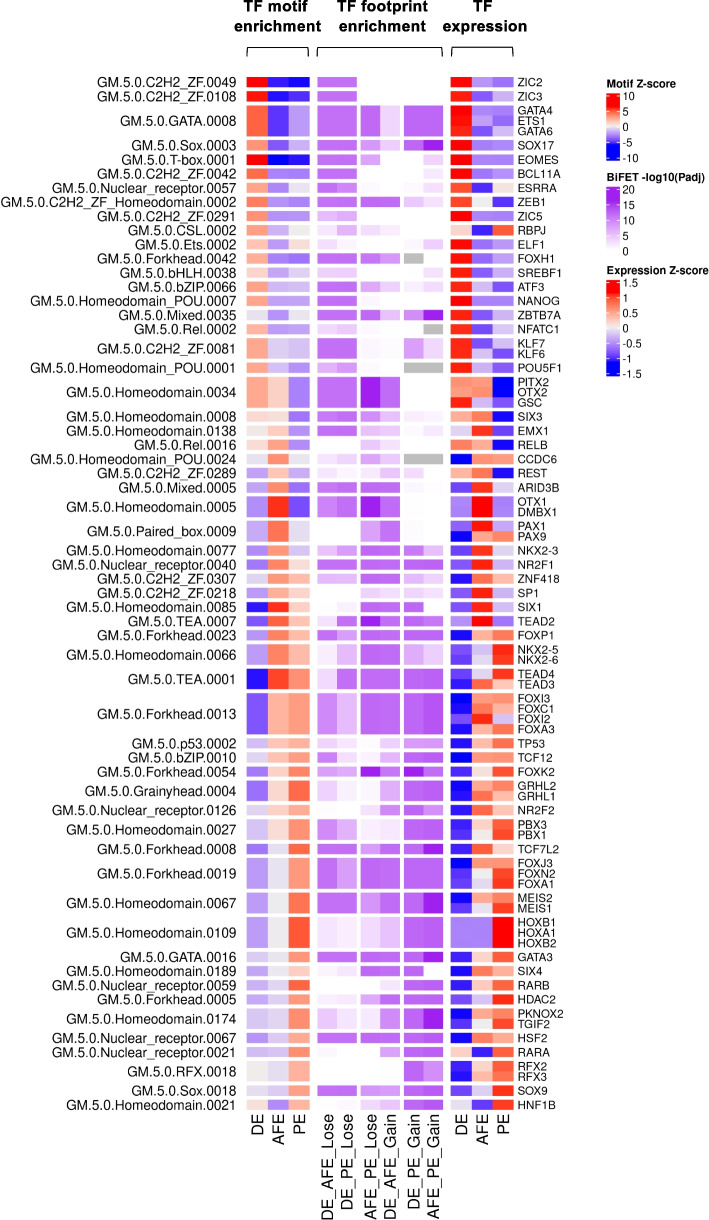
Chromatin accessibility and expression data allow the inference of cell type-specific transcription factor activity. Heatmaps showing the results of the integrated analysis of cell type-specific TF activity. The TF motifs here reported were selected based on maelstrom Z-score (heatmap on the left), BiFET adjusted *p*-value (heatmap in the middle), and TF expression (heatmap on the right), and on the correlation between these measures (see Methods). The motifs spanning multiple rows are associated with multiple TFs having expression correlated with enrichment; see also Additional File 8

The significant sequence conservation of the DARs we observed in the transition from DE to AFE (d5 -RA) and PE (d5 +RA) (Fig. 3A) suggests that such regions might be involved in regulating the differentiation process, possibly via the binding with protein regulators. Given the well-known role of transcription factors (TFs) in establishing transcriptional networks responsible for proper cell differentiation, we decided to investigate the putative TF binding profile of DARs. To this end, we used the maelstrom tool [67] to perform a differential motif enrichment analysis revealing which known TF motifs are specifically enriched in cell type-specific accessible regions. In parallel, the BiFET tool [68] was employed to identify TF footprints (FP: less accessible regions within highly accessible regions where a TF motif is found) [69] enriched in the DARs found in each differential accessibility contrast (Additional File 8: Table S5). The results of the maelstrom and of the BiFET analyses were integrated by selecting the TF motifs whose differential enrichment trend correlates with the corresponding FP enrichment profile and with the TF expression during the differentiation. This way, we identified a set of transcription factors, most of which are known regulators of DE, AFE or PE differentiation, which are differentially active among the three cell types (Fig. 4). Notably, among the TFs with PE-specific activity, we found several known regulators of PE differentiation such as FOXA1, FOXA2, NKX2-5, GATA3, PAX9, MEIS1, MEIS2, HOXA1, and HOXB1/2 (Fig. 4), supporting the idea that RA signaling regulates the accessibility of chromatin regions that are functionally relevant to PE commitment. As expected, among the motifs enriched in regions gaining accessibility in PE, we also found two DR5 type Retinoic Acid Responsive Elements (RAREs), whose enrichment correlates with the expression of RARA and RARB, two Retinoic Acid receptors (RARs) that are activated after the binding with RA and mediate the cellular response to this morphogen.

### Epigenetic characterization of regulatory elements reveals functional chromatin state changes between DE and PE stages

To further define and complement the epigenetic landscape of DE to PE differentiation, we performed ChIP-Seq analysis of H3K4me_3_, H3K27me_3_, H3K4me_1_, and H3K27ac histone marks (HM) on chromatin isolated from DE and PE cells. In addition to showing a clear agreement between biological replicates, the hierarchical clustering of samples based on ChIP-Seq read coverage also revealed that the two cell types have distinct HM profiles (Additional File 9: Fig. S4A). The proper HM distribution was confirmed by evaluating the DE and PE HM depositions around (± 3 kb) the TSSs of protein-coding genes, stratified based on the presence (or absence) of ATAC-Seq peaks, and around the summits of ATAC-Seq peaks located outside promoter regions (Additional File 9: Fig. S4B and C). H3K4me_3_ and H3K27ac show a bimodal distribution centered on the TSS, with a greater occupancy at sites where the chromatin is open both in DE and in PE (Additional File 9: Fig. S4B); differentially accessible TSSs display a clear and concordant change in the H3K27ac signal. Similarly, the H3K27me_3_ and H3K4me_3_ deposition at TSSs depends on chromatin accessibility dynamics, with a clear drop in the signal that is evident only at open TSS sites with no significant change in accessibility between DE and PE (Additional File 9: Fig. S4B). As expected, the HMs that were predominantly found within non-promoter ATAC-Seq peaks were H3K4me_1_ and H3K27ac, whose deposition positively correlates with the differential chromatin accessibility between DE and PE, concordantly with their well-established role as markers of regions with enhancer activity [70] (Additional File 9: Fig. S4C).

**Fig. 5:**
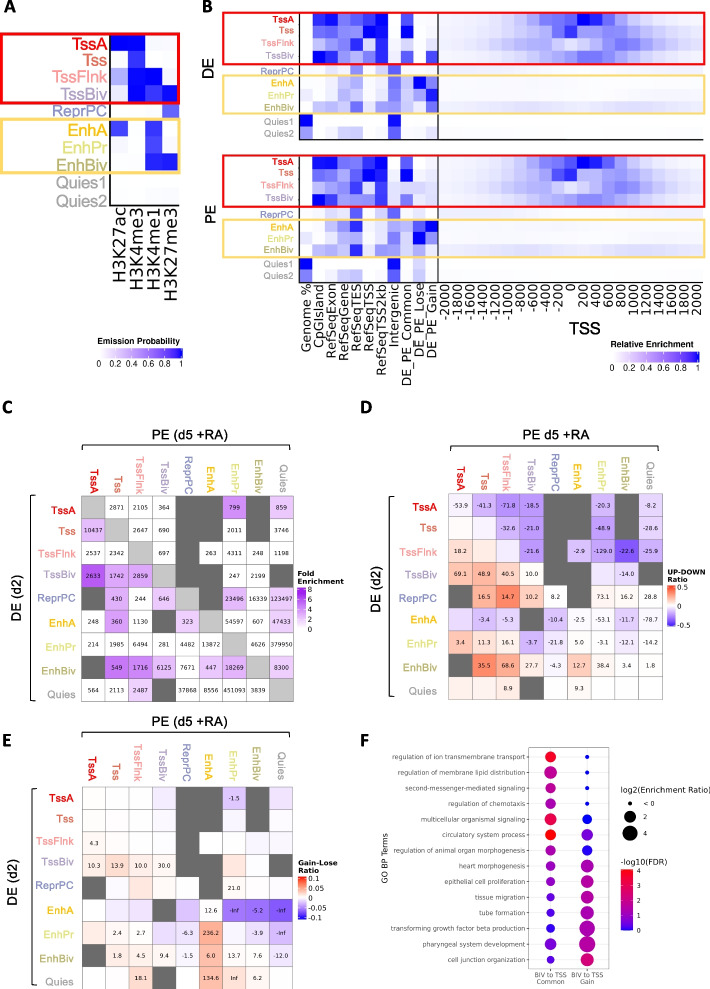
ChIP-Seq analysis reveals functional chromatin state changes between DE and PE stages. **A** Emission probabilities of the 10-state ChromHMM model. Each row represents a chromatin state and reports the frequency of occurrence of each HM in that state. Red and orange boxes indicate promoter and enhancer states, respectively. **B** Heatmaps showing the fold enrichment of each ChromHMM state for different genomic features (left panel) and at fixed positions relative to TSS (right panel) in DE and PE cells. The fold enrichments are calculated as the ratio between observed and expected number of genomic bins for each overlap, except for the Genome % column, which reports the percentage of genomic bins occupied by each state. The color intensities in the left panel are normalized within each column between its minimum value (white) and its maximum value (blue), while those in the right panel are normalized between the minimum value (white) and the maximum value (blue) of the whole matrix. Red and orange boxes indicate promoter and enhancer states, respectively. **C** Heatmap showing how many genomic bins transition from a chromatin state to another in the differentiation from DE to PE, as well as the fold enrichment of each transition (see Methods). Only cells corresponding to transitions with fold enrichment > 1.5 are colored, the color intensity being proportional to the fold enrichment. Poorly represented transitions (< 200 bins) are masked using dark grey color. **D** Heatmap showing the chromatin state transitions that are enriched in upregulated (red) or downregulated (blue) nearest genes. The color intensity is proportional to the difference between the number of upregulated and downregulated nearest genes, divided by the total number of nearest genes, while the digits within each cell correspond to the –log10(adjusted *p*-value) of the enrichment, with a – sign when the enrichment is towards downregulated genes. **E** Heatmap showing the chromatin state transitions that are enriched in Gain (red) or Lose (blue) ATAC-Seq peaks. The color intensity is proportional to the difference between the number of Gain and Lose overlapping peaks, divided by the total number of bins involved in the transition, while the digits within each cell correspond to the –log10(adjusted *p*-value) of the enrichment, with a – sign when the enrichment is towards Lose peaks. **F** Dot plot showing the GO BP terms enriched among the genes whose promoter transition from a bivalent state in DE (TssBiv, EnhBiv) to a TSS state in PE (TssA, Tss, TssFlnk) and overlap with a Common or Gain ATAC-Seq peak. The plot reports only a set of the significantly enriched categories (FDR < 0.05 in at least one class of TSS), selected in order to reduce redundancy

Taking advantage of the well-known distinct epigenetic signature of different functional genomic elements [70], we annotated the epigenome of DE and PE cells based on the presence of specific HM combinations (chromatin states) using the ChromHMM software [71]. We selected a 10-state model as the one which better and more concisely describes the meaningful combinations between the HMs under investigation (Fig. 5A); the human genome was segmented into 200 bp bins and each of such intervals was annotated with the states found in DE and PE. Based on the function that is commonly associated to known HM combinations [72] and on their overlap with annotated functional regions (Fig. 5B), we renamed the model states to: TssA (active/acetylated Promoter), Tss (Promoter), TssFlnk (Tss flanking region), TssBiv (bivalent promoter), ReprPC (Polycomb-repressed), EnhA (active/acetylated enhancer), EnhPr (primed enhancer), EnhBiv (bivalent enhancer), Quies1 and Quies2 (quiescent regions with no histone marks, fused into Quies state in subsequent analyses). Looking at how the genomic distribution of functional chromatin states changes in the transition from DE to PE, we observed a clear decrease in the number of genomic regions repressed by Polycomb and occupied by active enhancers, and an increase in primed enhancer occupancy (Additional File 9: Fig. S4D). Interestingly, by evaluating the overlap between chromatin states and ATAC-Seq peaks, we found that, while a significant number of Common peaks is located within regions with promoter-specific histone mark combinations, DARs are less frequently found in such regions and are enriched in enhancer-related signatures (Fig. 5B and Additional File 9: Fig. S4E), in line with the previously discussed genomic distribution of accessible regions. Furthermore, DARs are more frequently found in a quiescent state in the cell type in which the chromatin is less accessible (Additional File 9: Fig. S4E), indicating that, as expected, chromatin opening and closing events are accompanied by a concomitant change in histone mark deposition. To note, we also found that DE bivalent promoters are enriched in Gain peaks, suggesting that the activation of genes controlled by these promoters in the DE-PE transition might also be the result of an increase in chromatin accessibility. We then sought to characterize the chromatin state change dynamics in the transition between the DE and PE stages. To this end, for each transition between two distinct chromatin states observed in the differentiation process, we evaluated its enrichment with respect to the same change in the opposite direction (see Methods) [73] (Fig. 5C). The most relevant state changes emerging from this analysis are those going from bivalent or Polycomb-repressed states in DE to active and primed promoters and enhancers in PE. This is in line with the well-known gradual resolution of bivalent chromatin domains [74] during cell differentiation. We also observed a strong transition from active enhancers to quiescent states, which is concordant with the high number of intergenic regions losing accessibility in the differentiation from DE to PE, and a significant shift from deacetylated to acetylated promoters (Fig. 5C). To further validate the robustness of our model and to identify functional genomic regions associated to changes in gene expression and chromatin accessibility, we compared the state transitions with the expression of nearby genes (Fig. 5D) and evaluated the dynamics of overlapping ATAC-Seq peaks (Fig. 5E). As expected, those genes which are found in the proximity of genomic regions transitioning from an active to a repressed state tend to be downregulated, while transitions from repressive and quiescent states to active states are enriched in upregulated nearby genes (Fig. 5D). The state transitions showing the greatest overlap with Gain DARs are those leading to the formation of active and primed enhancers, while the regions in which such elements are lost are enriched in Lose DARs (Fig. 5E). Notably, the transition to a primed enhancer state displayed a strong association with an increase in nearby gene expression and chromatin accessibility, in line with a recent report showing that enhancers can activate the expression of nearby genes also in absence of the H3K27ac mark in mESCs [75]. This effect on nearby gene expression was not observed when the transition to primed enhancers started from quiescent chromatin, except for those cases in which it was accompanied by an increase in chromatin accessibility (Additional File 9: Fig. S4F). We also observed a positive correlation between increased accessibility and transition from a bivalent state to an active promoter (Fig. 5E). The genes controlled by such promoters showed an enrichment towards development-related functional categories, especially those whose TSS overlaps with a Gain peak, which displayed a specific involvement in the pharyngeal system development (Fig. 5F). This evidence strongly supports our model and suggests that, during PE differentiation, the chromatin transitions by silencing or selecting regulatory elements, most of which with enhancer signatures.

Finally, to elucidate the role of TFs in driving the PE development via binding to differentially accessible DNA sequences, we performed a FP enrichment analysis on both Gain and Lose DARs after stratifying them based on the overlap with different state transitions (Additional File 9: Fig. S4G). In addition to confirming the importance of the differentially active regulators reported in Fig. 4, this analysis also highlighted some differences in the TF binding profile between enhancer and promoter regions – e.g. the TSSs that become active and more accessible in PE are almost exclusively enriched in basic Helix-Loop-Helix (bHLH) TF binding (Additional File 9: Fig. S4G).

## Transcription factor regulatory network inference classifies TFs based on RA responsiveness and on RARA-mediated direct activation

Given the central role of the RA in PE specification, we sought to elucidate the molecular dynamics underlying this process. To this end, we conducted a ChIP-Seq experiment to characterize the binding profile of the Retinoic Acid Receptor alpha (RARA) in AFE (d5 -RA) and PE (d5 +RA) conditions, testing whether the RA directly influences RAR-RXR complex binding and epigenetic modifications during PE differentiation. Our analysis revealed 97 binding sites, and the presence of RARE motifs in approximately 60% of them underscores the specificity of the ChIP-Seq experiment.

**Fig. 6:**
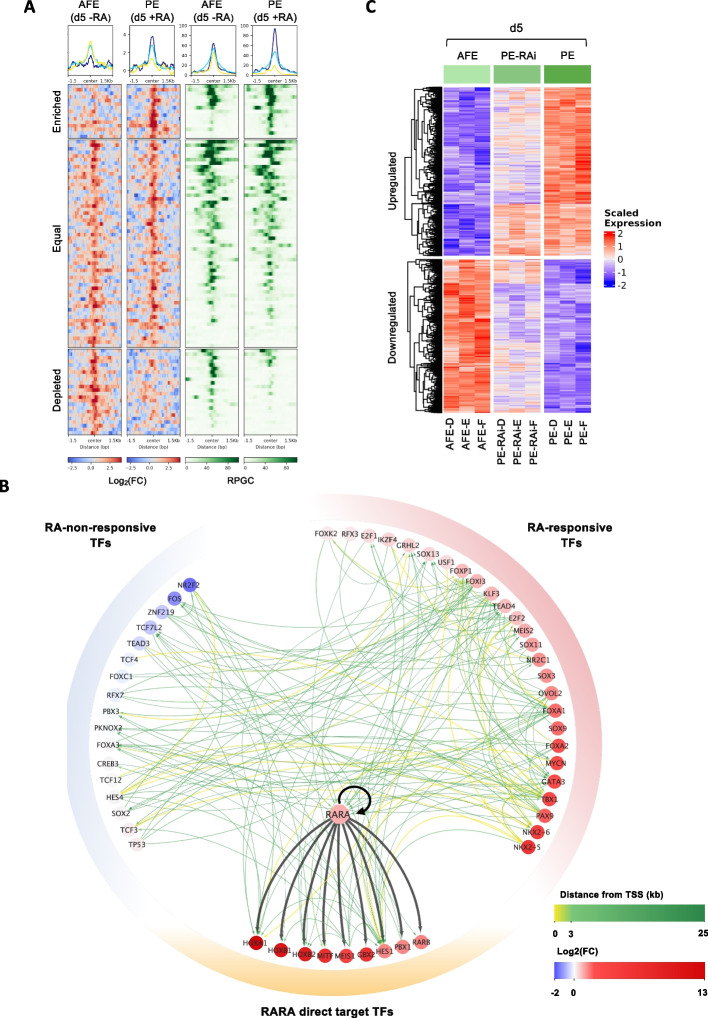
Multi-omics data integration allows to infer a PE-specific transcription factor network. **A** Heatmaps showing RARA ChIP-Seq and ATAC-Seq signal, measured in AFE (d5 -RA) and PE (d5 +RA) cells, within 3 kb-long regions centered on the summits of Enriched, Equal, and Depleted RARA-ChIP-Seq peaks. ChIP-Seq Signal was calculated as log2-transformed fold change of the RPGC values of IP over input, with a bin size of 50 bp. ATAC-Seq Signal was calculated on merged replicates as RPGC values with a bin size of 50 bp. Summary plots reporting the position-specific average signal calculated for each cell type and peak category are shown on top. **B** PE-specific TF-TF activation network. Nodes represent TFs that are specifically active and upregulated in PE (d5 +RA) with respect to DE (d2). Thin directed edges indicate the presence of a Gain ATAC-Seq peak harboring a source TF-specific FP located less than 25 kb from the TSS of the target TF; their color reflects the minimum distance between the target TSS and a Gain peak with a source-specific FP. Thick directed black edges connect RARA to its TF targets, identified via ChIP-Seq. Node color intensity is proportional to the log2(FC) in the expanded AFE vs PE comparison. See also Additional File 12. **C** Heatmap showing the expression in AFE (d5 -RA), PE-RAi (d5 +RA +AGN193109), and PE (d5 +RA) samples from Differentiation_2 experiment (see Methods) of the TFs belonging to the TFN, stratified based on their dependence on RA. Hierarchical clustering within each group is also shown. The expression values reported in the heatmap correspond to row-scaled (Z-score) rlog-transformed count data with batch effect correction

Interestingly, more than half of the identified peaks, labeled as “Equal”, showed no significant change in ChIP-Seq signal between AFE and PE (|log2FC|< 1), while the ones exhibiting increased or decreased binding in the presence of RA were defined as “Enriched” (log2[FC] > 1) or “Depleted” (log2[FC] < -1), respectively (Fig. 6A, left panel, and Additional File 10: Table S6**)**. By looking at the genomic distribution of the peaks, we observed that half of those belonging to the group “Equal” fall within promoters, while those which are Enriched or Depleted in PE are preferentially localized outside of these regions (Additional File 11: Fig. S5A).

By leveraging ATAC-Seq data, we found that RARA binds to regions where chromatin is accessible, both in AFE and PE, with the intensity of the ChIP-Seq signal positively correlated with that of ATAC-Seq (Fig. 6A). These observations align with the proposed model, wherein the presence of RA triggers the transcriptional activity of pre-existing RARA-RXRA complexes already bound to the chromatin [76]. Among the peaks identified in PE (d5 +RA), we found two enhancers located in proximity to the HOXA1 and HOXB1/B2 genes (Additional File 11: Fig. S5B). These RA-responsive enhancers become accessible between AFE (d5 -RA) and PE (d5 +RA), they harbor a conserved DR5 type RARE FP and are known to regulate the expression of these genes in both mice and humans [9, 77–80]. We also identified DR5-containing peaks at an alternative transcription start site of the RARA gene and within the promoter of the RARB gene (both genes induced during the PE differentiation) (Additional File 11: Fig. S5B), supporting the idea that RA can induce transcriptional positive feedback during the differentiation by directly inducing the transcription of the genes encoding its receptors. Unfortunately, the lack of available antibodies for ChIP-Seq prevented the identification of RARB binding sites, though it is likely that also this gene plays a significant role within the network.

Leveraging on the high-throughput data produced in this study, we inferred a predictive transcription factor network (TFN) that can be used as a tool to experimentally validate the contribution of each transcription factor in the PE development. To this aim, we used the ANANSE tool [81], which exploits RNA-Seq, ATAC-Seq, H3K27ac ChIP-Seq, and known TF motifs to build a differential regulatory network between two different cell types. This differential network was used to identify the set of transcription factors having the greatest influence on the transcriptional changes leading to PE specification (Additional File 11: Fig. S5C). Among them, we found TBX1 and HES1, two TFs active in PE [82–84] that did not emerge from our previous enrichment analyses. We then integrated the network with the TF footprint analysis and with the RARA ChIP-Seq data (Fig. 6B and Additional File 12). We stratified the TFs in three categories based on their expression profiles in the AFE (d5 -RA) and PE (d5 +RA) and on the binding of RARA (Additional File 10: Table S6): “RA-responsive” which exhibited induced expression in the presence of Retinoic Acid, but no evidence of direct RARA transcriptional activation, “RARA direct target” which were induced by the addition of RA and directly regulated by RARA, and “RA-non-responsive”, which showed no induction in the presence of RA (Fig. 6B). It is important to note that we cannot exclude that the RARA-RXR complexes bound on regions not in proximity of “RA-responsive” genes might still directly activate those genes during the PE differentiation through an unknown mechanism; alternatively, they could be directly regulated by RARB. Finally, we sought to validate the dependence of these TFs on Retinoic Acid by studying the transcriptome of cells differentiated in the presence of RA plus AGN 193109 (PE-RAi, d5 +RA +AGN193109), an antagonist of Retinoic Acid Receptors. Overall, the inhibition of RARs induced transcriptional changes, leading PE-RAi cells to shift towards the AFE condition (Fig. 6C). The suppression of RARs successfully counteracted the upregulation in the “RA-responsive” and “RARA direct target” gene categories. This effect was more consistently observed for the "RARA direct target" genes. In contrast, the genes in the “RA-non-responsive” category mainly displayed negligible and inconsistent effects, underscoring the robustness of our TFN (Additional File 11: Fig. S5D). Notably, when looking at changes in chromatin accessibility upon RA signal inhibition, we observed an analogue behavior to that of the transcriptome, with the chromatin accessibility in PE-RAi being intermediate between AFE and PE. Despite this behavior, when looking at the regions bound by RARA, the changes in PE-RAi were absent for the Equal peaks and less pronounced for the Enriched and Depleted peaks, despite following the same trend observed during differentiation (Additional File 11: Fig. S5E). This expected outcome further supports the notion that the RA induces the transcriptional competence of the RARA-RXRA complex, which is associated to accessible chromatin even in absence of RA, and that the induced transcription process might be responsible for an indirect reorganization of the chromatin.

## Discussion

The Pharyngeal Endoderm is a key cell type, given its ability to differentiate into organs and apparatuses whose proper formation is affected in several classes of human developmental syndromes displaying a complex spectrum of different phenotypes [6, 14]. Understanding the dynamics of human PE cell differentiation and identifying the factors driving this process is vital to understanding the pathogenesis of such diseases and to developing effective therapeutic strategies. Due to the early formation of PE in development (E21-28) and challenges in studying it in vivo, the molecular mechanisms behind human PE development remain largely unexplored. Cis-regulatory elements play a pivotal role during cell differentiation, given their ability to interact with tissue-specific TFs and orchestrate spatial and temporal gene expression. Sequence changes within these regions can significantly impact development by altering tissue-specific expression and causing phenotypic variation and diseases [85, 86]. Most of the focus in the field on generating and studying terminal cell types, like thymus or parathyroid cells, from in vitro differentiation protocols has left a gap in knowledge of the molecular networks of their precursor cell types, like PE. In this work, we have filled, in part, this important knowledge gap by developing a robust, reproducible in vitro platform for the derivation of transcriptionally homogenous human PE-like cells from hESCs through a defined, multi-step protocol involving the addition of known signaling agonists, including RA. We have demonstrated that RA is necessary and sufficient to induce the activation of a PE-specific transcriptional network via a dynamic and progressive remodeling of chromatin structure. Our PE-like cells have been deeply characterized and profiled based on their epigenetic and transcriptional signatures and resemble their mouse in vivo counterparts. By combining transcriptomic, chromatin accessibility, and histone mark analysis, we have outlined the stepwise gene regulation dynamics underlying PE differentiation and identified a subset of putative cis-regulatory elements that are likely to be bound by PE-specific TFs. Interestingly, we have found that most of the chromatin accessibility changes happen outside of promoters, in regions losing and acquiring enhancer-specific histone marks; conversely, at the promoter level, the prominent effect is a change in the histone mark signatures rather than a change in chromatin openness. We have then investigated the binding profile of RARA both in the presence and in the absence of RA. The occurrence of RARA binding in the AFE (d5 -RA) condition, within accessible chromatin regions, corroborates the existing knowledge about the RAR-RXR complex's mechanism of action, which activates transcription only in the presence of RA. This is particularly significant as it underscores the complex interplay between chromatin accessibility and transcription factor activity during development. Interestingly, among the RARA targets, we found RARB and an alternative RARA isoform, suggesting a potential positive feedback mechanism where RA stimulates the transcription of its own regulators. By combining the high-throughput data we generated, we have modeled the activation of a PE-specific transcriptional program by predicting a transcription factor network. This TFN was stratified based on the response of TFs to the presence of RA, and the ChIP-Seq data enabled us to pinpoint RA's direct targets. It is crucial to acknowledge the inherent nature of our TFN as a predictive model. As with any predictive tool, it primarily serves to identify potential candidates for further investigation. It facilitates a more comprehensive exploration of transcriptional regulation mechanisms, allowing us to propose hypotheses based on the TFN's outputs, and then rigorously test these hypotheses through experimental studies.

## Conclusions

In this study, we developed a new in vitro platform for the derivation of human PE-like cells and used computational genomics to elucidate the transcriptomic and epigenetic dynamics during human PE differentiation. While we have profiled the genomic landscape of PE cells, it is important to emphasize that the current study is descriptive in nature. Our work provides a list of putative candidates whose function will be experimentally validated in future studies. Such studies will be necessary to move beyond description and towards elucidating the biological mechanisms that underlie the observed genomic features. Our findings not only provide a foundational base for further mechanistic experiments to validate predicted interactions and establish cause-and-effect relationships but also offer a robust framework for investigating crucial, yet unexplored, molecular functions and identifying new players in PE development. Finally, the PE-like cells developed in our study serve as a developmental intermediary, offering the potential for the in vitro production and engineering of pharyngeal organs, a step with significant implications for precision medicine.

## Methods

### Cell culture conditions

H9 hESCs (purchased by WiCell, Cell line name WA09) were routinely propagated in feeder-free conditions in mTeSR1 on Matrigel coated (Corning, cat. n. 354,230) cell culture plates, following the manufacturer instructions and tested monthly for mycoplasma contamination. Undifferentiated hESCs were propagated and passed at least 3 times after thawing and plated for differentiation when at 80% of confluence. Cells were maintained in culture and expanded at high quality with particular care to avoid any spontaneous differentiation, which would confound downstream differentiation. The day before the induction of the differentiation, hESCs were washed twice in PBS (Cat. n. Gibco 10,010–023), dissociated with Accutase (Innovative Cell Technologies, Cat. n. AT104), plated in mTeSR1 + Rock Inhibitor (Y27632, 5uM) to promote cell survival and incubated for 12 h at 37 °C, 5% CO_2_ for 12 h. The day after, 30% confluent hESCs were induced to differentiate into definitive endoderm (DE), by incubation with Medium A (Gibco cat. n. A30621-01) for 24 h and with Medium B (Gibco cat. n. 30,624–01) for the following 24 h at 37 °C, 5% CO_2_. The third day DE was patterned into AFG by the addition of CDM2 medium with A-83–01, 1uM and DM3189, 250 nM, as we previously reported [20]. The composition of CDM2 basal medium was as follows: 50% IMDM (+ GlutaMAX, + HEPES, + Sodium Bicarbonate; Gibco, 31,980–097) + 50% F12 (+ GlutaMAX; Gibco, 31,765–092) + 1 mg/mL polyvinyl alcohol (Sigma, P8136-250G) + 1% v/v concentrated lipids (Gibco, 11,905–031) + 450 µM monothioglycerol (Sigma, M6145) + 0.7 µg/mL insulin (Roche, 1,376,497) + 15 µg/mL transferrin (Roche, 652,202) and incubated at 37 °C, 5% CO_2_. From day four to day seven, Retinoic Acid 200 nM was added at the CDM2 medium with A-83–01 1 uM, DM3189 250 nM, and the medium was changed every 24 h. Cells collected at day 5 were called AFE (d5 -RA) or PE (d5 +RA), depending on the presence of RA in the medium. This first differentiation experiment was called Differentiation_1. To inhibit the response to Retinoic Acid during differentiation, we performed an independent differentiation experiment in triplicate (Differentiation_2) to derive, in addition to AFE (d5 -RA) and PE cells (d5 +RA), a cellular type named PE-RAi (d5 +RA +AGN193109), obtained by adding the pan-Retinoic Acid Receptor antagonist AGN193109 (Tocris, 5758) in a concentration of 50 nM to the CDM2 medium with A-83–01, 1uM and DM3189, 250 nM, on day3 of differentiation, and collecting the cells at day 5. To note: the stability of Retinoic Acid might change based on the lot number; for this reason, we suggest performing a titration when a new lot number is used prior to starting the differentiation, since the amount needed might vary between lot numbers (the range is usually between 50-200 nM).

### RNA isolation and quantitative-RT PCR (qRT-PCR)

Total RNA from hESCs, DE, AFE, and PE cells was isolated by collecting cells using TRI Reagent (Zymo Research R2053), followed by column purification and DNAse treatment using Direct-zol RNA MiniPrep Kit (Zymo Research R2053), and quantified by Nanodrop (Thermo Scientific). RNA (0.5–1.0 μg) was reverse transcribed using PrimeScript Reagent Kit (Takara, RR037B) according to manufacturer’s instructions for quantitative RT-PCR analyses. Quantification analyses were carried out using PowerUp SYBR-Green MasterMix (Thermo Fisher Scientific, A25741). The sequences of the oligonucleotides used for the different amplifications are reported in Additional File 13: Table S7.

### Immunofluorescence staining

Cells were fixed with 4% paraformaldehyde for 15 min at room temperature, washed three times in PBS for 5 min each, incubated for permeabilization with in 0.5% Triton-X 100 PBST for 15 min at room temperature, washed three time with PBST for 5 min each, and then incubated with blocking solution of 5% bovine serum albumin (Sigma-Aldrich, A7906-100G) in PBST overnight at 4˚C. Cells were incubated with Primary antibodies overnight at 4˚C (see Additional File 13: Table S7 for dilutions**)** in 1% bovine serum albumin PBST. The day after, cells were washed three times in PBST for 5 min each, incubated with appropriate secondary fluorophore-conjugated antibodies (Invitrogen) diluted in a 1% bovine serum albumin solution in PBST at room temperature for 2 h repaired from light. Cells were washed three times for five minutes protected from light in PBS. Slides were mounted with ProLong™ Diamond Antifade Mountant with DAPI (Thermo Fisher Scientific, P36962) and images were produced with a EVOS M7000 microscope.

### TBX1-flag-mRUBY cell line generation

Specific guides to cut the genomic region encompassing exon 9 and 3’ untranslated region (UTR) of TBX1 isoform c were selected. The FLAG tag and the mRuby reporter gene were inserted in the genome of H9 embryonic stem cells through homologous recombination by using a construct containing the in frame 3xFLAG-tag and mRuby gene separated by a self-cleaving T2A peptide flanked by homology regions. The guides were cloned into a pSpCas9-GFP (PX458) and the donor vector into a pUC19 plasmid. The plasmids were delivered via electroporation using the P3 Primary Cell 4D-Nucleofector® X kit (Lonza, V4XP-3024). Cells were then plated and cultured following the protocol described above, supplementing the culture media with Rock Inhibitor (Y27632, 5uM) to increase the cell survival rate. The cells successfully electroporated were selected based on GFP expression by flow cytometry and plated at low density to obtain single cell clones. Colonies originated from single cells were then moved to single wells of 48 well plates and genotyped via PCR. Positive clones were finally sequenced to confirm the absence of mutations and the proper insertion of the construct.

### Flow cytometry analysis

Wild-type and TBX1-flag-mRUBY cells were detached with TrypLE™ Express (gibco, 12,605–010), centrifuged for 5 min at 500 g, and washed twice in 500µL of FACS Buffer. Cells were then resuspended in 250 µL of FACS Buffer and reduced to a single-cell suspension by passing them through the 35 µm cell strainer caps of flow cytometry tubes (Falcon). Cells were then scanned with a FACSAria II flow cytometer (BD Biosciences) and data were analyzed with FlowJo Mac version 10.7.1 (Tree Star).

### Bulk RNA-Seq experiments

RNA library preparations and sequencing reactions were conducted at GENEWIZ, Inc/Azenta US, Inc. (South Plainfield, NJ, USA). 1 ug of RNA, extracted from three biological replicates of hESCs, DE, AFE (d5 -RA), and PE (d5 +RA) cells from Differentiation_1, and from three biological replicates of AFE (d5 -RA), PE (d5 +RA), and PE-RAi (d5 +RA +AGN193109) cells from Differentiation_2, was quantified using Qubit 2.0 Fluorometer (Life Technologies, Carlsbad, CA, USA) and RNA integrity was checked using Agilent TapeStation 4200 (Agilent Technologies, Palo Alto, CA, USA). RNA sequencing libraries were prepared using the NEBNext Ultra RNA Library Prep Kit for Illumina using manufacturer’s instructions (NEB, Ipswich, MA, USA). Briefly, mRNAs were initially enriched with Oligo(dT) beads. Enriched mRNAs were fragmented for 15 min at 94 °C. First strand and second strand cDNA were subsequently synthesized. cDNA fragments were end-repaired and adenylated at 3’ ends, and universal adapters were ligated to cDNA fragments, followed by index addition and library enrichment by PCR with limited cycles. The sequencing library was validated on the Agilent TapeStation (Agilent Technologies, Palo Alto, CA, USA), and quantified by using Qubit 2.0 Fluorometer (Invitrogen, Carlsbad, CA) as well as by quantitative PCR (KAPA Biosystems, Wilmington, MA, USA). The sequencing libraries were clustered on a single lane of a flow cell. After clustering, the flow cell was loaded on an Illumina HiSeq 4000 instrument according to the manufacturer's instructions. The samples were sequenced using a 2 × 150 bp paired-end configuration. Two different sequencing experiments were performed for cells from Differentiation_1 and Differentiation_2. Image analysis and base calling were conducted by the HiSeq Control Software (HCS). Raw sequence data (.bcl files) generated from Illumina HiSeq was converted into fastq files and de-multiplexed using Illumina's bcl2fastq v2.17 software. One mismatch was allowed for index sequence identification. On average, ~ 33 and ~ 22 million read pairs were obtained for each sample from Differentiation_1 and Differentiation_2, respectively.

### Bulk RNA-Seq data analysis

Adapter sequences and poor quality ends were removed using the Trimmomatic v0.39 software [87] with parameters *ILLUMINACLIP:/path/to/adapter:2:30:10:1:true SLIDINGWINDOW:20:15 MINLEN:36*. To produce coverage tracks, reads first were mapped to human GRCh38 genome and GENCODE v35 transcriptome [88] using STAR v2.7.6a software [89], with parameters *–peOverlapNbasesMin 10 –outSAMstrandField intronMotif –outFilterIntronMotifs RemoveNoncanonical –outSAMattrIHstart 0 –outSAMtype BAM SortedByCoordinate*; SAMtools v1.11 merge tool [90] was used to pool together read alignments from biological replicates, thus producing a single pooled BAM file for each condition; deepTools v3.5.1 [91] bamCoverage tool was then employed to convert pooled BAM files to bigWig files while removing reads mapping to ENCODE Blacklist regions [92], with RPKM normalization and the genomic bin size set to 10 bp. Salmon v1.3.0 tool [93] was employed to quantify transcript expression, producing isoform-level transcripts per million (TPM) values from a full decoy transcriptome index created using the GENCODE v35 transcriptome and the hg38 genome. Tximport v1.18.0 R package [94] was employed to obtain gene-level TPMs and estimated counts. Such counts were used for the differential gene expression analysis, performed using the DESeq2 v1.30.0 R package [95], after removing genes with TPM < 1 in at least 10 samples (when comparing hESCs, DE, AFE, and PE cells from Differentiation_1), or 7 samples (when comparing AFE and PE cells using samples from Differentiation_2 [Differentiation_2 AFE vs PE comparison] and from both Differentiation experiments [expanded AFE vs PE comparison]). For each contrast performed using only Differentiation_1 samples, differential gene expression analysis with independent filtering was run twice, by setting the *lfcThreshold* for the Wald test either to 0 or to 0.58, thus producing a relaxed and a strict set of differentially expressed genes (the FDR threshold was set to 0.01 in both cases); only genes with an average TPM > 1 in at least one of the two conditions under comparison were retained. For the Differentiation_2 and expanded AFE vs PE comparisons, a covariate representing the different sequencing and differentiation experiments was introduced in the design formula; only for the expanded comparison the FDR threshold was set to 0.05 to increase the sensitivity of the detection of RA-responsive genes, a choice justified by the use of a greater number of samples in the differential expression analysis. Apeglm [96] method was employed for log2(Fold Change [FC]) shrinkage. Regularized-log (rlog) transformation was applied to count data for subsequent clustering analysis and to produce the heatmaps showing the expression of the DEGs identified via the strict test in Differentiation_1 samples and the gene expression measured in Differentiation_2 samples; in the latter case, a batch effect correction was applied using the “removeBatchEffect” function from the limma v3.46.0 R package [97]. The principal component analysis (PCA) plot was drawn using the DESeq2 “plotPCA” function; the sample-to-sample euclidean distance heatmap was produced using the pheatmap v1.0.12 R package (available at https://CRAN.R-project.org/package=pheatmap). The UpSet plot showing the intersections between DEGs identified in each contrast was drawn using the UpSetR v1.4.0 R package [98]. The gene expression heatmaps were generated using the ComplexHeatmap v2.6.2 [99] R package.

### Comparison of Bulk RNA-Seq samples with mouse scRNA-Seq samples

The count matrix relative to the scRNA-Seq data from embryonic mouse foregut endoderm produced by Han and colleagues [34] was downloaded from the Gene Expression Omnibus GEO archive (GSE136689) [100]. Counts were transformed to counts per million values (CPM), and average CPM values were calculated for each gene in each endodermal cell cluster. For each cluster, we retrieved all the markers as well as the top 10 transcription factor markers from the original publication. The similarity of each cluster with our bulk RNA-Seq samples was assessed by computing the Spearman correlation coefficient between the log10-transformed CPM values of its top transcription factor (TF) markers and the log10-transformed TPM values of their human counterparts. Mouse-Human orthology relationships were retrieved from Ensembl 101 database [101]. The Spearman correlation matrices and the HOX gene expression heatmaps were plotted using the ComplexHeatmap R package. For the comparison with the scRNA-Seq data produced by Magaletta and colleagues [63], we retrieved the top 10 TF markers of each cluster from the original publication. Due to the unavailability of metadata detailing the assignment of cells to the clusters identified by the authors, we evaluated the similarity solely based on the number of TF markers specific to each cluster that exhibited adequate expression (TPM > 5) in our PE cells.

### scRNA-Seq experiment

For single cell RNA-Seq experiments, two batches were prepared: Batch_1 included hESCs (d0), DE (d2), and PE (d5 +RA) cells, while Batch_2 contained DE (d2), AFE (d3), AFE (d5 -RA), and PE (d5 +RA) cells. Cells were washed twice with CDM2 medium to remove dead cells and detached using Accutase. Cells were collected and counted using Countess® II Automated Cell Counter. 500,000 cells for each condition were collected in a PBS-0.04%BSA buffer and processed according to the 10X Genomics Single Cell Protocols Cell Preparation Guide (https://assets.ctfassets.net/an68im79xiti/56DlUZEsVOWc8sSG42KQis/35cbcf6dcd4b0c0196263ee93815b0ae/CG000053_CellPrepGuide_RevC.pdf). For each cell type, 7000 cells for Batch_1 and 20,000 cells for Batch_2 were loaded per lane on the 10 × Genomics Chromium platform, with the goal of capturing 2500 cells and 10,000 cells, respectively. Cells were then processed for cDNA synthesis and library preparation using 10X Genomics Chromium Version 2 chemistry (catalog number 120234) as per the manufacturer’s protocol. cDNA libraries were checked for quality on the Agilent 4200 Tape Station platform and their concentration was quantified by KAPA qPCR. Libraries were sequenced using an Illumina HiSeq 4000 instrument to a depth of, at a minimum, 70,000 reads per cell. For hESCs, sequencing data were previously produced from our lab [102] and are available in the GEO repository with the accession number GSE157475.

### scRNA-Seq data analysis

Illumina base call files were converted to FASTQ files using the Cell Ranger v2.0 program (1). FASTQ files were then aligned to the hg19 human reference genome using Cell Ranger. The Scanpy v1.7.2 Python package [103] was used for subsequent analyses.

We combined cells from two batches and all the samples into a single “anndata” object. Batch_1 contained 6115 hESCs cells, 2566 DE (d2) cells and 2525 PE (d5 +RA) cells, while Batch_2 contained 10,919 DE (d2) cells, 11,422 AFE (d3) cells, 16,051 AFE (d5 -RA) cells and 10,059 PE (d5 +RA) cells. Quality control metrics, including the number of detected genes per cell, the total counts per cell and the percentage of counts belonging to mitochondrial genes were calculated using the function “scanpy.pp.calculate_qc_metrics”. We first filtered out low-quality cells that expressed fewer than 13,000, 20,000 and 15,000 counts for hESCs, DE (d2), and PE (d5 +RA), respectively, for Batch_1; 13,000, 10,000, 10,000 and 15,000 counts for DE (d2), AFE (d3), AFE (d5 -RA), and PE (d5 +RA) for Batch_2. These thresholds were chosen based on the distribution of the total counts for each sample. We also excluded cells that expressed more than 9000 genes (which would imply doublets) or that expressed more than 10% mitochondrial genes (indicative of dead cells in this dataset) [104]. Finally, we filtered out cells with less than 2500 expressed genes using the function “scanpy.pp.filter_cells” and genes expressed in less than 10 cells using the function “scanpy.pp.filter_genes”. After quality control, we obtained 4917 hESCs cells, 1320 DE (d2) cells, and 1284 PE (d5 +RA) cells for Batch_1; 8133 DE (d2) cells, 9219 AFE (d3) cells, 9241 AFE (d5 -RA) cells, and 6166 PE (d5 +RA) cells for Batch_2. Next, normalization was performed by dividing raw counts by the library counts sum and multiplying by a factor of 100,000, using the function “scanpy.pp.normalize_total” with *target_sum* parameter set to 100,000. After log normalization, the highly variable genes were selected with the function “scanpy.pp.highly_variable_genes”, with *max_mean*, *min_mean* and *min_disp* parameters set to 5, 0.0125, and 0.5, respectively, obtaining 2918 genes. From this set we removed ribosomal genes, finally obtaining 2904 highly variable genes. The total counts and the percentage of mitochondrial counts were regressed out as potential confounding factors with the function “scanpy.pp.regress_out”. Genes were then scaled to zero mean and unit variance, clipping maximum values to 10 (“max_value” parameter). A principal component analysis was performed on the scaled matrix with the function “scanpy.tl.pca”, using the “arpack” singular value decomposition solver, and a k-nearest neighbor graph was computed with the function “scanpy.pp.neighbors”.

Next, we removed batch effects using Batch Balanced KNN (BBKNN, bbknn Python module version 1.5.1) [105] with the pre-computed PCA as dimensionality reduction method and using as batch key for integration the two batches of data, containing 7521 and 32,759 cells. Dimensionality reduction through the Uniform manifold approximation and projection (UMAP) [64] algorithm was performed with the function “scanpy.tl.umap” using the batch-corrected gene expression matrix. Cell clustering was performed with the Leiden algorithm [65] using the function “scanpy.tl.leiden” on the BBKNN corrected matrix, using a range of values of the “resolution” parameter between 0.2 and 0.5. Differentially expressed genes between clusters were identified with the function “scanpy.tl.rank_genes_groups” using the t-test with overestimated variance; for each group, the top 50 DEGs were chosen based on the Z-score returned by this function.

We found that the PE (d5 +RA) cell type was the last one splitting in sub clusters, as shown in a clustering tree computed using the R package “clustree” version 0.5.0. Moreover, we obtained 1107 differentially expressed genes (FDR < 0.05, |log2FC|> 1) between the two PE (d5 +RA) sub-clusters emerging at resolution = 0.5, which do not contain any classical PE marker genes, again witnessing its homogeneity. All single-cell RNA-Seq plots were also generated using Scanpy.

### Retrieval of transcription start sites and promoter regions

TSSs of protein-coding transcripts with annotated 5’UTR were retrieved from Refseq v109.20211119 curated annotation [106] and from the “upstream1000.fa” file provided by the UCSC Genome Browser [107] (available at https://hgdownload.soe.ucsc.edu/goldenPath/hg38/bigZips/). The hg38 genomic coordinates of such TSSs were extended by 3000 bp both upstream and downstream to obtain a set of promoter regions. These promoters were assigned to their corresponding GENCODE protein-coding genes using the BEDTools intersect tool. Promoters of protein-coding genes that do not produce polyadenylated transcripts were not kept for further analyses; such genes were defined as non-expressed genes (TPM < 1 in at least 10 Bulk RNA-Seq samples) that do not overlap with any poly(A) feature from PolyASite 2.0 database [108] and GENCODE annotation (available at https://www.gencodegenes.org/human/release_35.html).

### ATAC-Seq experiments

ATAC-Seq library preparation and sequencing reactions were conducted at GENEWIZ, Inc/Azenta US, Inc. (South Plainfield, NJ, USA). DE (d2), AFE (d5 -RA), and PE (d5 +RA) live cell samples from Differentiation_1, and AFE (d5 -RA), PE (d5 +RA), and PE-RAi (d5 +RA +AGN193109) live cell samples from Differentiation_2 (two biological replicates for each condition) were thawed, washed, and treated with DNAse I (Life Tech, Cat. #EN0521) to remove genomic DNA contamination. Live cell samples were quantified and assessed for viability using a Countess Automated Cell Counter (ThermoFisher Scientific, Waltham, MA, USA). After cell lysis and cytosol removal, nuclei were treated with Tn5 enzyme (Illumina, Cat. #20,034,197) for 30 min at 37 °C and purified with Minelute PCR Purification Kit (Qiagen, Cat. #28,004) to produce tagmented DNA samples. Tagmented DNA was barcoded with Nextera Index Kit v2 (Illumina, Cat. #FC-131–2001) and amplified via PCR prior to a SPRI Bead cleanup to yield purified DNA libraries. The sequencing libraries were clustered on one lane of a flow cell. After clustering, the flow cell was loaded on an Illumina HiSeq 4000 instrument according to the manufacturer's instructions. The samples were sequenced using a 2 × 150 bp PE configuration. Two different sequencing experiments were performed for cells from Differentiation_1 and Differentiation_2. Image analysis and base calling were conducted by the HiSeq Control Software (HCS). Raw sequence data (.bcl files) generated from Illumina HiSeq was converted into fastq files and de-multiplexed using Illumina's bcl2fastq v2.20 software. One mismatch was allowed for index sequence identification. On average, ~ 98 and ~ 40 million read pairs were obtained for each sample from Differentiation_1 and Differentiation_2, respectively.

### ATAC-Seq data analysis

Sequencing adapters and low-quality bases were trimmed using the Trimmomatic v0.39 software with parameters *ILLUMINACLIP:/path/to/adapter:2:30:10:1:true SLIDINGWINDOW:20:15 MINLEN:36*. Preprocessed reads were then aligned to the hg38 genome using Bowtie2 [109] with parameters *–wrapper basic-0 –fr -X 2000*. Aligned reads were filtered using SAMtools to keep only concordant primary alignments having a minimum mapping quality of 30. PCR or optical duplicates were marked using Picard v2.25.1 tool (available at https://broadinstitute.github.io/picard/) and removed. Reads mapping to mitochondrial DNA and to unplaced contigs were filtered out. Aligned reads were also shifted as in [110] using the deepTools alignmentSieve tool with the *–ATACshift* parameter. After this shift, reads falling in ENCODE Blacklist regions were removed using BEDTools v2.30.0 pairToBed tool [111]. Read alignments from biological replicates were pooled together using SAMtools merge. deepTools bamCoverage tool was then employed to convert BAM files (both from individual and pooled replicates) to bigWig files with Reads Per Genome Coverage (RPGC) normalization and the genomic bin size set to 10 bp (for track visualization) and to 50 (for coverage heatmaps).

MACS2 v2.2.7.1 callpeak tool [112] was used to identify open chromatin regions in each replicate, with parameters *-f BAMPE –call-summits -g hs –keep-dup all*. Peaks identified by MACS2 in all the samples were used to determine a consensus peak set using the “dba” function from the DiffBind v3.0.13 R package [113], setting the *minOverlap* parameter to 2. Reads mapping in 201 bp intervals centered on consensus peak summits were counted using the “dba.count” function, with the *filter* parameter set to 0; counts were normalized using full library size with the “dba.normalize” function. PCA was drawn using the “dba.plotPCA” function; Pearson correlation coefficient values, calculated on the normalized read counts between each pair of samples using the “dba.plotHeatmap” function, were employed to draw a sample-to-sample distance matrix using the pheatmap R package. Only consensus peaks called by MACS2 in both replicates of at least one condition were employed to draw the Venn diagram, produced using the BioVenn v1.1.3 R package [114]. Differential accessibility analysis was performed for each contrast with the “dba.analyze” function, setting the underlying method to DESeq2; a paired design, justified by the timing of sample preparation and sequencing, was employed only for the DE vs PE contrast. For each A vs B contrast, we identified three classes of 201 bp peaks:

- Common peaks, called by MACS2 in both replicates of A and/or B and with DiffBind FDR > 0.01 and/or absolute log2(FC) < 1;
- Lose peaks, called by MACS2 in both replicates of A and with DiffBind FDR < 0.01 and log2(FC) < -1;
- Gain peaks, called by MACS2 in both replicates of B and with DiffBind FDR < 0.01 and log2(FC) > 1.

The heatmap showing the clustering of differentially accessible regions (DARs) was produced using the ComplexHeatmap R package. PhyloP basewise conservation scores derived from Multiz alignment of 100 vertebrate species [115] were retrieved for DARs and Common peaks using the GenomicScores v2.2.0 R package [116].Heatmaps and profile plots of Differentiation_1 ATAC-Seq signal around the TSSs of protein-coding genes and the summits of RARA ChIP-Seq peaks were drawn by applying the deepTools computeMatrix, plotHeatmap and plotProfile tools to the previously produced BigWig files with 50 bp resolution. Heatmaps and profile plots of Differentiation_2 ATAC-Seq signal were drawn for the DARs identified by comparing Differentiation_2 AFE (d5 -RA) and PE (d5 +RA) samples and for the RARA binding regions identified via ChIP-Seq analysis.

The protein-coding promoter chromatin accessibility status was evaluated by searching for overlaps between protein-coding promoters and consensus ATAC-Seq peaks using BEDTools intersect. Non-promoter peaks were identified based on the absence of overlap with any TSS ± 3 kilobases (kb) region derived from RefSeq and UCSC knownGene annotation. The proximity of any consensus ATAC-Seq peak to protein-coding gene TSSs was evaluated using the BEDTools closest tool.

### Transcription factor motif and footprint analyses

The TF motifs used in the present work are those composing the non-redundant, clustered gimme.vertebrate.v5.0 database, which is available within the GimmeMotifs v0.17.0 analysis framework [67]. To identify differentially enriched motifs among DE, AFE (d5 -RA), and PE, we first collected all DARs and calculated the average of the normalized counts across the biological replicates for each condition; such mean accessibility measurements were then log2-transformed and subsequently centered by subtracting the mean of the log2-transformed values across the three conditions. The resulting table of scaled read counts was provided to the GimmeMotifs maelstrom tool, which was run with the *–no-filter* option. This tool combines different motif enrichment methods to calculate, for each TF motif, a set of condition-specific combined Z-scores, each one representing the enrichment of the motif among the condition-specific accessible regions.

TF footprints (FP) were individually identified for DE, AFE (d5 -RA), and PE conditions by applying the 2017–04-27 version of the PIQ tool [117] to pooled BAM files, using the “pairedbam2rdata.r” script to convert them to internal binary format, setting the purity score threshold to 0.7 and using the gimme.vertebrate.v5.0 motif file, after converting it to JASPAR format [118] with the UniversalMotif v1.8.3 R package (available at https://bioconductor.org/packages/universalmotif/), as input motif database for the “pwmmatch.r” script; only motifs belonging to TF with average TPM > 5 in at least condition were used in this analysis.

FP enrichment analysis was performed using the BiFET tool [68]. Specifically, for each A vs B differential accessibility contrast, BiFET was employed to evaluate the enrichment of FPs identified in B among the Gain peaks and the enrichment of FPs identified in A among the Lose peaks, using the Common peaks as background loci in both cases. The normalized read counts and the GC content of each ATAC-Seq consensus peak, which are employed by BiFET for bias correction, were calculated using DiffBind and HOMER v4.11.1 tools [119], respectively. “findOverlaps” function from the GenomicRanges v1.42.0 R package [120] was used to find the FPs overlapping consensus peaks.

For the integrated analysis of TF activity, only motifs with an absolute maelstrom Z-score ≥ 2 in at least one condition, a BiFET adjusted *p*-value < 0.001 in at least one set of DARs and an average transcription factor gene TPM > 5 in at least one cell type were initially selected. Furthermore, only TF genes found to be differentially expressed in at least one contrast were employed. BiFET adjusted *p*-values were converted to –log10(adjusted *p*-values), after replacing 0 values with 1 × 10^–16^ to avoid infinite numbers; the average of these transformed *p*-values was computed for each cell-type specific set of DARs, thus obtaining a FP enrichment score for each condition. Z-scores of log2-transformed average TPMs along the cell types were also computed to obtain a set of condition-specific expression Z-scores for each TF. The final set of cell type-specific TF motifs was obtained by selecting only motifs whose maelstrom Z-scores are positively correlated (Pearson correlation coefficient > 0.5) with the FP enrichment scores and with the expression Z-scores. The heatmap showing the enrichment of these motifs was drawn using the ComplexHeatmap R package.

### ChIP-Seq experiments

ChIP experiments were performed on chromatin extracts according to the manufacturer's protocol (MAGnify ChIP, Life Technologies Cat. n. 492,024). For the immunoprecipitation reaction of RARα, 60 µg of sheared chromatin from AFE (d5) and PE (d5 +RA) differentiated cells was used, while for each other immunoprecipitation reaction, 10ug of sheared chromatin from DE (d2) and PE (d5 +RA) differentiated cells was used (two biological replicates per ChIP experiment). Sheared chromatin was incubated O.N. with 5 μg of anti- H3K27me_3_ (Abcam Cat. n. ab6002), H3K4me_3_ (Active Motif Cat. n. 39,159), H3K27ac (Active Motif Cat. n. 39,133), H3K4me_1_ (Abcam ab8895), or RARα (Diagenode Cat. n. C15310155) antibodies (Additional File 13: Table S7). ChIP-Seq library preparation and sequencing reactions were conducted at GENEWIZ, Inc/Azenta US, Inc. (South Plainfield, NJ, USA). Immunoprecipitated (IP) and input DNA samples were quantified by Qubit 2.0 Fluorometer (Invitrogen, Carlsbad, CA) and the DNA integrity was checked with 4200 TapeStation (Agilent Technologies, Palo Alto, CA, USA). NEBNext Ultra DNA Library Preparation kit was used following the manufacturer’s recommendations (Illumina, San Diego, CA, USA). Briefly, the ChIP DNA was end-repaired and adapters were ligated after adenylation of the 3’ ends. Adapter-ligated DNA was size selected, followed by clean up, and limited cycle PCR enrichment. The ChIP library was validated using Agilent TapeStation and quantified using Qubit 2.0 Fluorometer as well as real time PCR (Applied Biosystems, Carlsbad, CA, USA). The sequencing libraries were multiplexed and clustered on one lane of a flow cell. After clustering, the flow cell was loaded on an Illumina HiSeq 4000 instrument according to the manufacturer’s instructions (Illumina, San Diego, CA, USA). Sequencing was performed using a 2 × 150 bp PE configuration. Image analysis and base calling were conducted by the HiSeq Control Software (HCS). Raw sequence data (.bcl files) generated from Illumina HiSeq was converted into fastq files and de-multiplexed using Illumina's bcl2fastq v2.17 software. One mismatch was allowed for index sequence identification. On average, ~ 49 and ~ 27 million read pairs were obtained for each histone modification and RARA ChIP-Seq sample, respectively.

### Histone modification ChIP-Seq data analysis

The preprocessing, alignment and post-alignment filtering of reads, as well as the generation of bigWig files with RPGC normalization, were performed as in the ATAC-Seq data analysis, except for the read alignments shift, which was skipped. 50 bp resolution bigWig files for individual replicates of immunoprecipitated samples were given as input to deepTools multiBigwigSummary to compute average RPGC scores for 10 kb genomic bins; deepTools plotCorrelation was employed on the resulting output file to produce the Pearson correlation matrix and the hierarchical clustering of samples. In addition, we compared the 50 bp resolution pooled coverage tracks of IP and input samples using deepTools bigwigCompare to generate BigWig files reporting the log2(FC) of the IP signal over the input for each 50 bp genomic bin. deepTools computeMatrix, plotHeatmap and plotProfile tools were applied to these files to draw heatmaps and profile plots of ChIP/input signal around the TSSs of protein-coding genes and the summits of ATAC-Seq consensus peaks. For the former analysis, TSSs were stratified based on the overlap with ATAC-Seq peaks, evaluated using BEDTools intersect after replacing the boundaries of the DiffBind consensus peaks with those of the corresponding merged MACS2 peaks.

Chromatin state discovery was performed using the ChromHMM v1.22 software. As a first step, all the BAM files were binarized with the BinarizedBam module, using a bin size of 200 bp. Concatenated model learning was conducted with the LearnModel module, using the input samples as control data to adjust the binarization threshold locally. This module was employed to build models with a number of states ranging from 6 to 16. We decided to focus on a model with 10 states for the subsequent analyses, since it delivered a compact and meaningful representation of the main chromatin states that can be produced with the 4 histone marks under analysis. The LearnModel module produced 200 bp chromatin state calls for DE and PE cell types. OverlapEnrichment module was employed to compute, both for DE and PE chromatin state annotations, the fold enrichment relative to a set of genomic features derived from the RefSeq annotation and to the ATAC-Seq consensus peaks, classified based on the DE vs PE contrast. The fold enrichment of chromatin states relative to the neighborhood of TSSs derived from RefSeq annotation was computed with the NeighborhoodEnrichment module. The relationship between accessible regions and chromatin states was investigated by using BEDTools intersect to assign ATAC-Seq consensus peaks to the 200 bp genomic bins containing their summits. Protein-coding TSSs were assigned to their corresponding bins using the “findOverlaps” function from the GenomicRanges R package.

The state transition enrichment analysis was inspired by a work by Fizier and colleagues [73]. Specifically, we first calculated the number of 200 bp bins involved in each possible chromatin state transition from DE to PE; for each transition, we also calculated the expected number of transitioning bins as the average of the number of transitions obtained after shuffling the state calls 1000 times; we then divided the observed counts by the expected counts to compute an enrichment score for each transition, thus controlling for the state coverage; finally, fold enrichment values were obtained by dividing the enrichment score of each transition by the enrichment score of the transition having opposite direction, thus controlling for the overall similarity between the two states involved in the transition.

For each genomic bin, the nearest expressed protein-coding gene (average TPM > 1 in DE and/or in PE) with a TSS within a distance of 50 kb, if any, was identified using the “distanceToNearest” function from the GenomicRanges R package. To test the association between chromatin state transitions and deregulation of nearby genes, for each state transition we calculated the number upregulated, downregulated and non-differentially expressed genes in the proximity of the bins undergoing the transition (*UP*_*T*_, *DOWN*_*T*_, *NO*_*T*_) and of all the other bins (*UP*_*O*_, *DOWN*_*O*_, *NO*_*O*_), used as controls (also transitions between identical states were employed in this analysis). Then, three Fisher’s exact tests were performed for each transition (the numbers within the square brackets representing a row of a 2 × 2 contingency table), obtaining a set of *p*-values (adjusted using the Benjamini–Hochberg procedure):

- *P*_*UPDOWN*_: [*UP*_*T*_, *DOWN*_*T*_] vs [*UP*_*O*_, *DOWN*_*O*_];
- *P*_*UP*_: [*UP*_*T*_, (*DOWN*_*T*_ + *NO*_*T*_)] vs [*UP*_*O*_, (*DOWN*_*O*_ + *NO*_*O*_)];
- *P*_*DOWN*_: [*DOWN*_*T*_, (*UP*_*T*_ + *NO*_*T*_)] vs [*DOWN*_*O*_, (*UP*_*O*_ + *NO*_*O*_)].

For each transition, we also computed:

- *RATIO*_*UPDOWN*_: $$\frac{\frac{{UP}_{T}-{DOWN}_{T}}{{UP}_{T}+{DOWN}_{T}+{NO}_{T}}}{\frac{{UP}_{O}-{DOWN}_{O}}{{UP}_{O}+{DOWN}_{O}+{NO}_{O}}}$$;
- *RATIO*_*UP*_: $$\frac{\frac{{UP}_{T}}{{UP}_{T}+{DOWN}_{T}+{NO}_{T}}}{\frac{{UP}_{O}}{{UP}_{O}+{DOWN}_{O}+{NO}_{O}}}$$;
- *RATIO*_*DOWN*_: $$\frac{\frac{{DOWN}_{T}}{{UP}_{T}+{DOWN}_{T}+{NO}_{T}}}{\frac{{DOWN}_{O}}{{UP}_{O}+{DOWN}_{O}+{NO}_{O}}}$$.

A state transition was considered as enriched in nearby upregulated genes when *RATIO*_*UPDOWN*_ > 1, *RATIO*_*UP*_ > 1.2, *P*_*UPDOWN*_ < 0.05 and *P*_*UP*_ < 0.05 or enriched in nearby downregulated genes when *RATIO*_*UPDOWN*_ < 1, *RATIO*_*DOWN*_ > 1.2, *P*_*UPDOWN*_ < 0.05 and *P*_*DOWN*_ < 0.05.

The association between chromatin state transitions and differential chromatin accessibility was evaluated following a similar procedure, in which the number of upregulated genes was replaced by the number of bins with Gain peaks, the number of downregulated genes was replaced by the number of bins with Lose peaks and the number of non-differentially expressed genes was replaced by the number of genomic bins with no Gain or Lose peaks.

For the FP enrichment analysis of chromatin state-specific DARs, performed using BiFET, we focused on transitions enriched either in Lose or Gain peaks. Lose and Gain peaks were divided based on their chromatin state in DE and PE, respectively. For each class of Lose peaks, we evaluated the FP enrichment of downregulated TFs with an average DE TPM > 5. For each class of Gain peaks, we evaluated the FP enrichment of upregulated TFs with an average PE TPM > 5. In both cases, for each state-specific class of DARs, we used all the Common peaks with a corresponding chromatin state in DE and/or in PE as background regions.

The heatmaps showing the results of the enrichment analyses performed on chromatin state transitions and on state-specific DARs were drawn using the ComplexHeatmap R package.

### RARA ChIP-Seq analysis

The preprocessing, alignment and post-alignment filtering of reads, as well as the generation of bigWig files, were performed as in the histone modification ChIP-Seq data analysis. The HOMER makeTagDirectory and findPeaks tools [119] were employed to call peaks for each IP sample, comparing them with the corresponding input samples, setting the findPeaks *style* parameter to *factor* and keeping only peaks with peak score ≥ 20. A consensus peak set was generated using the DiffBind “dba” function, with the *minOverlap* parameter set to 1. Reads mapping in 401 bp intervals centered on consensus peak summits were counted using the “dba.count” function, with the *filter* parameter set to 0; counts were normalized using full library size with the “dba.normalize” function after subtracting input counts. For each consensus peak, log2(FC) of the PE normalized counts over the AFE normalized counts was calculated, and it was used to classify the peak as Enriched (log2[FC] ≥ 1), Depleted (log2[FC] ≤ -1) or Equal (|log2[FC]|< 1). deepTools computeMatrix, plotHeatmap and plotProfile tools were used to draw heatmaps and profile plots of ChIP/input signal around the summits of the consensus peaks. For each consensus peak, the closest protein-coding gene TSS was determined using the “distanceToNearest” function from the GenomicRanges R package. The GimmeMotifs scan tool was used to search for occurrences of the Retinoic Acid Responsive Element (RARE) motifs (GM.5.0.Nuclear_receptor.0020, GM.5.0.Nuclear_receptor.0021, GM.5.0.Nuclear_receptor.0036, GM.5.0.Nuclear_receptor.0053, GM.5.0.Nuclear_receptor.0059) within the consensus peaks.

### Functional enrichment and feature distribution analyses

All the Gene Ontology (GO) [32] Biological Process term enrichment analyses were performed using the Over-Representation Analysis (ORA) method available within the WebGestaltR v0.4.4 R package [121]. For each analysis we used a different reference set:

- ORA of DEG clusters: all the genes with average TPM > 1 in at least one of the DE, AFE (d5 -RA), and PE conditions;
- ORA performed on markers of embryonic mouse foregut endodermal cell type clusters with average TPM in our PE cells > 5: all the genes with average TPM > 5 in PE condition;
- ORA of protein-coding genes with a TSS transitioning from a bivalent state in DE (TssBiv or EnhBiv) to an active promoter state in PE (TssA, Tss or TssFlnk): all the protein-coding genes with average TPM > 1 in DE and/or PE.

WebGestaltR was employed to perform Gene Set Enrichment Analysis (GSEA) [61], using shrunken log2(FC) values as a ranking metric.

GREAT analysis [66] of each DAR cluster was performed employing the rGREAT v1.22.0 R package (available at https://github.com/jokergoo/rGREAT), using all the DARs and Common peaks as background regions.

The bar plots showing the genomic annotation of ATAC-Seq and ChIP-Seq peaks were produced using the ChIPseeker v1.26.0 R package [122], employing as a TxDb object the one provided by the TxDb.Hsapiens.UCSC.hg38.knownGene v3.10.0 R package (https://bioconductor.org/packages/release/data/annotation/html/TxDb.Hsapiens.UCSC.hg38.knownGene.html).

### Transcription factor network inference

ANANSE v0.3.0 + 3.g18995f0 software [81] was used to infer a transcription factor network (TFN) for DE and PE stages and to identify the key TFs in the PE specification process. First, the binding module was employed to predict transcription factor binding individually for each cell type, by providing it with the filtered BAM files obtained from ATAC-Seq and H3K27ac HM CHIP-Seq data, and using the ANANSE REMAP model v1.0 (available at https://zenodo.org/record/4768075/files/ANANSE.REMAP.model.v1.0.tgz), which includes average ChIP-Seq signal obtained from the ReMap database [123]; the default motif database (gimme.vertebrate.v5.0) was used in this step. The resulting output files were separately given as input to the network module, thus producing two GRNs, one for DE and one for PE. Finally, the influence module was used to calculate a differential GRN and to compute the influence scores for the transition from DE to PE, providing it with the DE and PE GRNs as the source and target networks, respectively, and with the results of the relaxed differential gene expression analysis performed on the DE vs PE contrast. To increase the number of possible TF-target interactions, which were subsequently filtered during the generation of the PE-specific TF-TF interaction network, the differential GRN was obtained using the top 1,000,000 edges of both source and target networks. To build the PE-specific TF-TF interaction network, the differential GRN was initially filtered to retain only the TF-TF interactions with a differential score > 0.7. To focus on factors specifically active in PE, we only kept interaction involving TFs having average TPM in PE > 5 and which met at least one of the following conditions:

- From the integrated TF activity analysis, the TF was found to be specifically active in the PE stage (all the TFs below TEAD2 in Fig. 4);
- From the FP enrichment analysis of chromatin state-specific DARs, the TF FPs were found to be enriched in at least one class of Gain peaks (BiFET *p*-value < 0.001);
- The TF was among the 30 top TFs based on ANANSE sumScaled influence score.

Finally, we only kept the interactions in which the source TF had a FP inside a Gain peak (DE vs PE contrast) located at less than 50 kb from the TSS of the target TF. The resulting network was imported in the Cytoscape v3.9.1 software [124], which was used to arrange and visualize it, after filtering out the interactions mediated by Gain peaks located at less than 25 kb from the target to better view the most relevant interactions. The network was integrated by incorporating the interactions between RARA and its TF targets identified via ChIP-Seq analysis. To qualify as a “RARA direct target”, a transcription factor had to be the nearest protein-coding gene relative to a RARA ChIP-Seq peak called in PE and had to exhibit upregulation in PE compared to DE. All the identified TF targets were already present in the network, except for GBX2, which was added to it. The remaining TFs were categorized as "RA-responsive" if they showed upregulation in the expanded AFE vs PE comparison, or as "RA-non-responsive" otherwise. Network nodes were annotated with the log2(FC) calculated in the expanded AFE vs PE comparison. The Cytoscape session file which also contains the interactions in the 25–50 kb range is provided as Additional File 12.

### Supplementary Information

Additional file 1. Supplementary figure S1.

Additional file 2. Supplementary table S1.

Additional file 3. Supplementary table S2.

Additional file 4. Supplementary table S3.

Additional file 5. Supplementary figure S2.

Additional file 6. Supplementary figure S3.

Additional file 7. Supplementary table S4.

Additional file 8. Supplementary table S5.

Additional file 9. Supplementary figure S4.

Additional file 10. Supplementary table S6.

Additional file 11. Supplementary figure S5.

Additional file 12. TF_network.cys.

Additional file 13. Supplementary table S7.

Additional file 14. Review history.

## Data Availability

Bulk RNA-Seq, single-cell RNA-Seq, ATAC-Seq, and ChIP-Seq data associated with this study are available in the GEO repository with the accession number GSE208319, https://www.ncbi.nlm.nih.gov/geo/query/acc.cgi?acc=GSE208319 [125]. Previously published sequencing data used in this study are available in the GEO repository with the accession numbers GSE136689 [126] and GSE157475 [127]. No other scripts and software were used other than those mentioned in the Methods section.
