# Supplementary material for: Transcriptional and epigenetic characterization of a new in vitro platform to model the formation of human pharyngeal endoderm
Source: Genome Biol. 2024 Aug 8;25:211. doi: 10.1186/s13059-024-03354-z (PMC11312149; doi:10.1186/s13059-024-03354-z)
Supplement: Supplementary file 1 — Additional file 1. Supplementary figure S1. [file 13059_2024_3354_MOESM1_ESM.pdf]

Figure S1

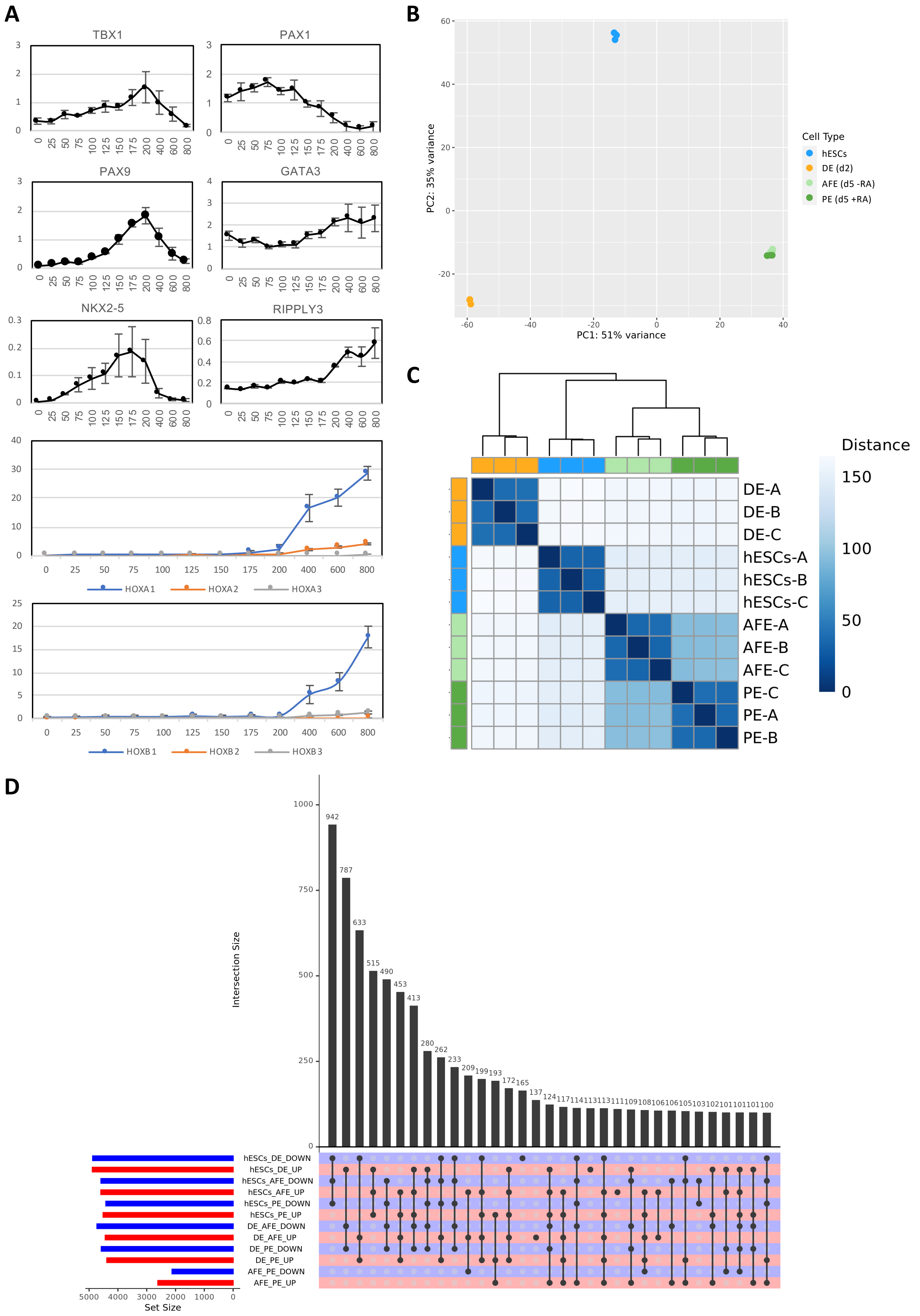
A
TBX1
PAX1
PAX9
GATA3
NKX2-5
RIPPLY3
HOXA1
HOXA2
HOXA3
HOXB1
HOXB2
HOXB3
B
PC1: 51% variance
PC2: 35% variance
Cell Type
hESCs
DE (d2)
AFE (d5 -RA)
PE (d5 +RA)
C
Distance
DE-A
DE-B
DE-C
hESCs-A
hESCs-B
hESCs-C
AFE-A
AFE-B
AFE-C
PE-C
PE-A
PE-B
D
Intersection Size
Set Size
hESCs_DE_DOWN
hESCs_DE_UP
hESCs_AFE_DOWN
hESCs_AFE_UP
hESCs_PE_DOWN
hESCs_PE_UP
DE_AFE_DOWN
DE_AFE_UP
DE_PE_DOWN
DE_PE_UP
AFE_PE_DOWN
AFE_PE_UP

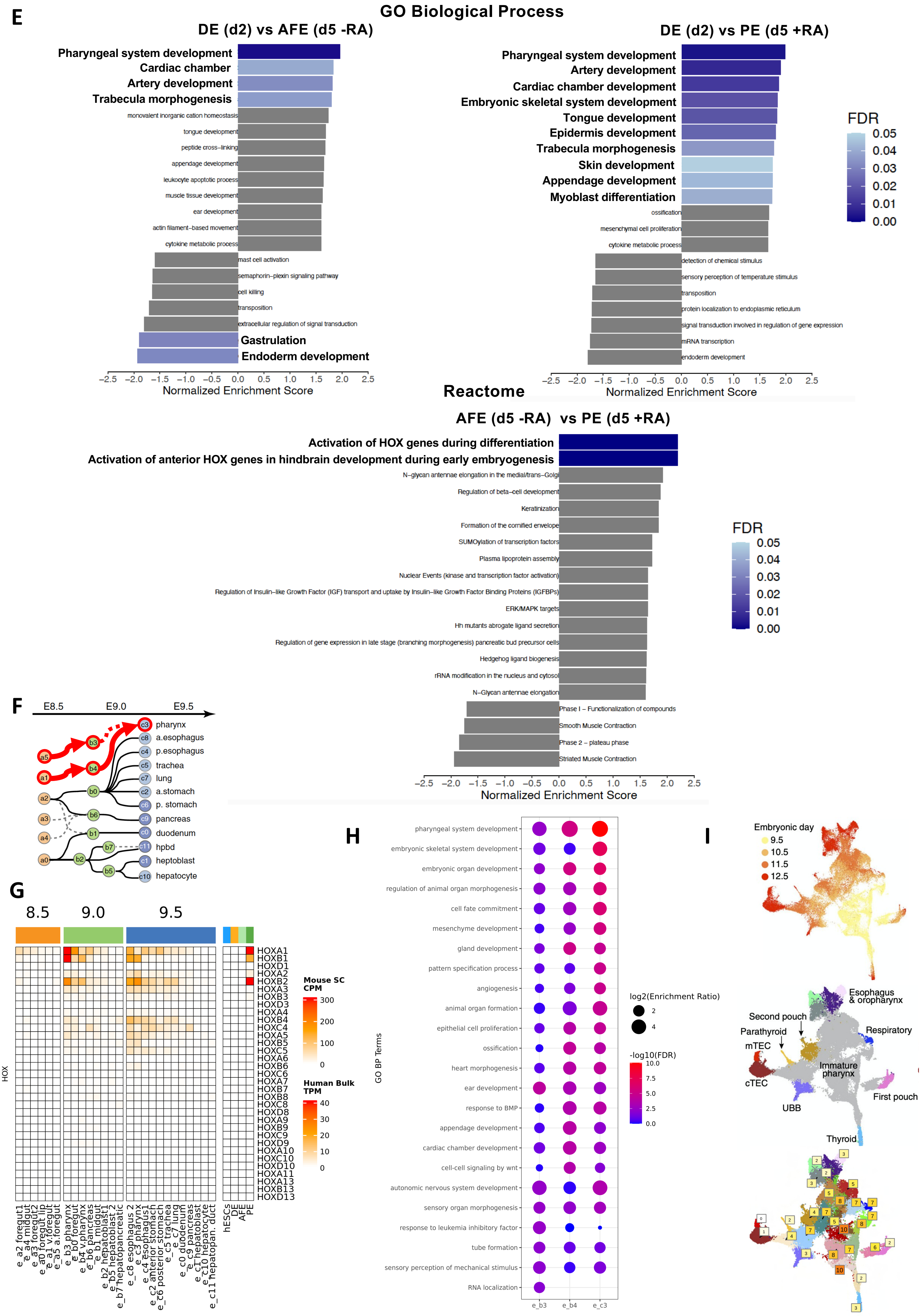
E
GO Biological Process
DE (d2) vs AFE (d5 -RA)
Pharyngeal system development
Cardiac chamber
Artery development
Trabecula morphogenesis
monovalent inorganic cation homeostasis
tongue development
peptide cross−linking
appendage development
leukocyte apoptotic process
muscle tissue development
ear development
actin filament−based movement
cytokine metabolic process
mast cell activation
semaphorin−plexin signaling pathway
cell killing
transposition
extracellular regulation of signal transduction
Gastrulation
Endoderm development
Normalized Enrichment Score
DE (d2) vs PE (d5 +RA)
Pharyngeal system development
Artery development
Cardiac chamber development
Embryonic skeletal system development
Tongue development
Epidermis development
Trabecula morphogenesis
Skin development
Appendage development
Myoblast differentiation
ossification
mesenchymal cell proliferation
cytokine metabolic process
detection of chemical stimulus
sensory perception of temperature stimulus
transposition
protein localization to endoplasmic reticulum
signal transduction involved in regulation of gene expression
mRNA transcription
endoderm development
Normalized Enrichment Score
FDR
Reactome
AFE (d5 -RA) vs PE (d5 +RA)
Activation of HOX genes during differentiation
Activation of anterior HOX genes in hindbrain development during early embryogenesis
N−glycan antennae elongation in the medial/trans−Golgi
Regulation of beta−cell development
Keratinization
Formation of the cornified envelope
SUMOylation of transcription factors
Plasma lipoprotein assembly
Nuclear Events (kinase and transcription factor activation)
Regulation of Insulin−like Growth Factor (IGF) transport and uptake by Insulin−like Growth Factor Binding Proteins (IGFBPs)
ERK/MAPK targets
Hh mutants abrogate ligand secretion
Regulation of gene expression in late stage (branching morphogenesis) pancreatic bud precursor cells
Hedgehog ligand biogenesis
rRNA modification in the nucleus and cytosol
N−Glycan antennae elongation
Phase I − Functionalization of compounds
Smooth Muscle Contraction
Phase 2 − plateau phase
Striated Muscle Contraction
Normalized Enrichment Score
FDR
F
E8.5
E9.0
E9.5
pharynx
a.esophagus
p.esophagus
trachea
lung
a.stomach
p. stomach
pancreas
duodenum
hpbd
heptoblast
hepatocyte
G
8.5
9.0
9.5
HOX
Mouse SC CPM
Human Bulk TPM
hESCs
DE
AFE
PE
H
GO BP Terms
pharyngeal system development
embryonic skeletal system development
embryonic organ development
regulation of animal organ morphogenesis
cell fate commitment
mesenchyme development
gland development
pattern specification process
angiogenesis
animal organ formation
epithelial cell proliferation
ossification
heart morphogenesis
ear development
response to BMP
appendage development
cardiac chamber development
cell-cell signaling by wnt
autonomic nervous system development
sensory organ morphogenesis
response to leukemia inhibitory factor
tube formation
sensory perception of mechanical stimulus
RNA localization
log2(Enrichment Ratio)
-log10(FDR)
e_b3
e_b4
e_c3
I
Embryonic day
9.5
10.5
11.5
12.5
Esophagus & oropharynx
Second pouch
Parathyroid
Respiratory
mTEC
Immature pharynx
cTEC
First pouch
UBB
Thyroid

Figure S1: Bulk RNA-Seq analysis of hESCs, DE (d2), AFE (d5 -RA), and PE (d5 +RA) cell types (related to Figure 1).

(A) RT-qPCR marker quantification from an *in vitro* differentiation to PE (d5) using increasing RA concentration (0-800nM). Data were normalized on PDGB expression and represent means ± SEM of three independent time course experiments. **(B)** Principal Component Analysis (PCA) plot showing the clustering of hESCs, DE (d2), AFE (d5 -RA), and PE (d5 +RA) samples based on their gene expression profile measured via Bulk RNA-Seq of biological triplicates. Principal component 1 (PC1) and 2 (PC2) were identified based on the rlog-transformed counts relative to the 500 most variable genes. **(C)** Sample-to-sample euclidean distance heatmap showing the similarity of hESCs, DE (d2), AFE (d5 -RA), and PE (d5 +RA) samples based on their gene expression profile measured via Bulk RNA-Seq of biological triplicates. Euclidean distances between rlog-transformed count profiles were also used to draw the dendrogram showing the hierarchical clustering of samples. **(D)** UpSet plot showing the number of significantly downregulated and upregulated genes (absolute log2[FC] significantly > 0, FDR < 0.01) for all the possible contrasts among hESCs, DE (d2), AFE (d5 -RA), and PE (d5 +RA) samples, along with the intersections of different sets consisting of at least 100 genes; see also Additional File 2. **(E)** Bar plot showing the top 20 functional categories based on absolute Normalized Enrichment Score (NES) calculated performing Gene Set Enrichment Analysis (GSEA) on three contrasts: DE (d2) vs AFE (d5 -RA) and DE (d2) vs PE (d5 +RA) (upper panel), for which enriched non-redundant GO Biological Processes are shown, and AFE (d5 -RA) vs PE (d5 +RA) (bottom panel), for which enriched Reactome categories are shown. Positive NESs stand for enrichment among upregulated genes, negative NESs stand for enrichment among downregulated genes. Different shades of blue are used to represent categories with FDR < 0.05, whose names are also highlighted; grey is used to represent categories with FDR ≥ 0.05. **(F)** Spatial and temporal developmental tree with lineage prediction of Endodermal Cells during mouse embryonic foregut development at E8.5, E9.0, and E9.5 (adapted from Han et al., 2020 [34]). The developmental trajectory of our PE (d5 +RA) cells based on the analysis shown in Fig. 1E is highlighted in red. **(G)** Heatmaps of HOX genes expression profile in the different mouse endodermal cell clusters (data from Han et al., 2020 [34]) at different developmental stages (left) and in HESCs, DE (d2), AFE (d5 -RA), and PE (d5 +RA) samples (right). Expression levels are shown as gene-level Transcript Per Million (TPM) values. (**H**) Dot plot reporting the results of the GO non-redundant BP term enrichment analyses performed on the markers identified in the endodermal cell clusters e_b3, e_b4, and e_c3 that are expressed with > 5 TPM in PE (d5 +RA) cells. Only the top 10 enriched terms found for each cluster are shown. (**I**) Comparison of PE (d5 +RA) cells with cells from mouse pharyngeal endoderm at E9.5, E10.5, E11.5, and E12.5. The UMAP visualization, adapted from Magaletta et al., 2022 [63], is colored by embryonic day (top panel), anatomical structures (middle panel), and Louvain clusters (lower panel), as defined in the original publication. For each Louvain cluster, the figure in the lower panel reports how many of the top 10 TF markers are expressed with TPM > 5 in PE (d5 +RA).
