# Supplementary material for: Transcriptional and epigenetic characterization of a new in vitro platform to model the formation of human pharyngeal endoderm
Source: Genome Biol. 2024 Aug 8;25:211. doi: 10.1186/s13059-024-03354-z (PMC11312149; doi:10.1186/s13059-024-03354-z)
Supplement: Supplementary file 5 — Additional file 5. Supplementary figure S2. [file 13059_2024_3354_MOESM5_ESM.pdf]

Figure S2

A

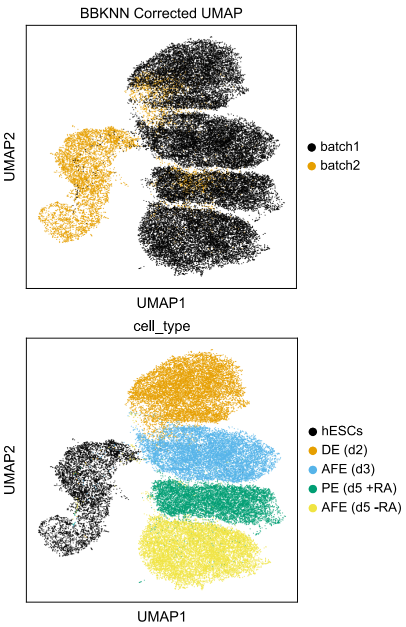

B

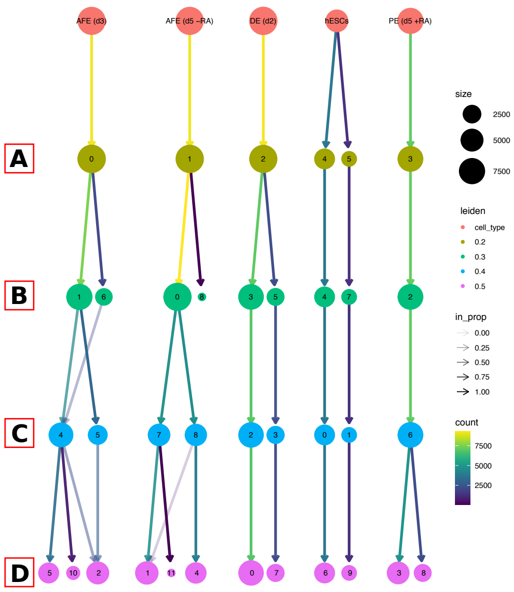

C

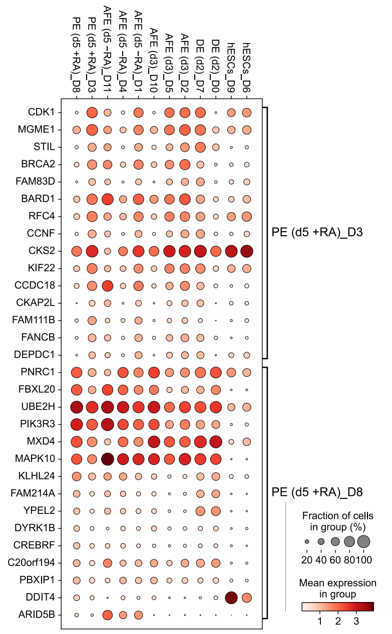

D

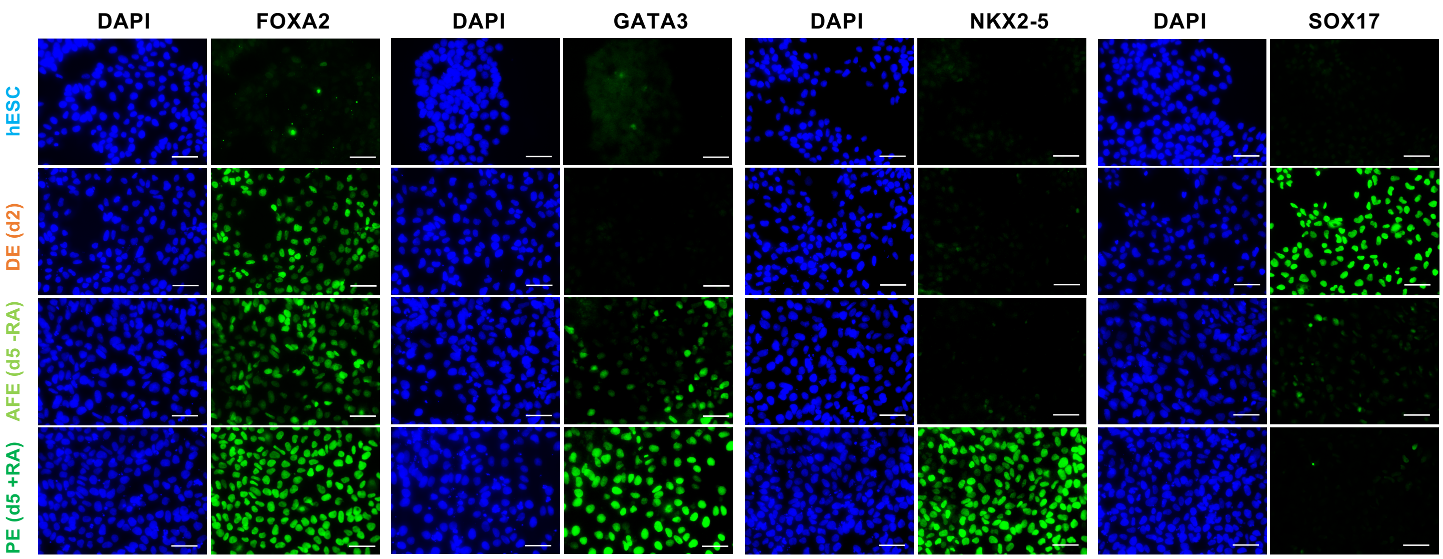

E

H9-WT

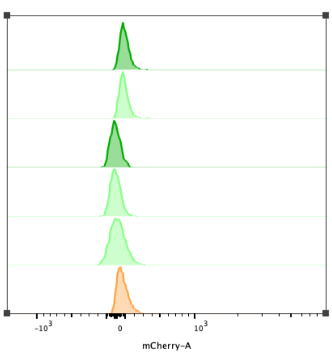

	Sample	Count	Freq. of Positive (%)
	PE (d5 +RA)	7934	0.3
	AFE (d5 -RA)	8123	0.1
	PE (d4 +RA)	3427	0.1
	AFE (d4 -RA)	6638	0
	AFE (d3)	7822	0
	DE (d2)	6453	0

H9-TBX1-mRUBY

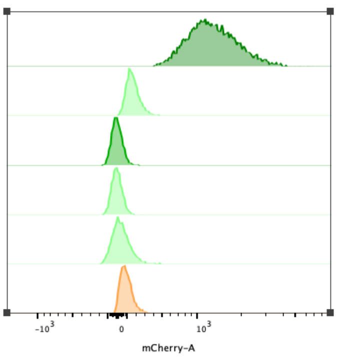

	Sample	Count	Freq. of Positive (%)
	PE (d5 +RA)	7666	**99.5**
	AFE (d5 -RA)	7984	0.7
	PE (d4 +RA)	7268	0.4
	AFE (d4 -RA)	7878	0.1
	AFE (d3)	7572	0.3
	DE (d2)	7307	0.1

Figure S2: Single-cell RNA-Seq analysis of hESCs, DE (d2), AFE (d3), AFE (d5 -RA), and PE (d5 +RA) cell types (related to Figure 2).
(**A**) UMAP plot showing the integration of the 2 experimental batches using BBKNN. (**B**) Clustering tree showing the splitting of the cell types (top level) at different values of the resolution parameter from the Leiden clustering algorithm. The dot size is proportional to the size of the cluster, the transparency of the arrows represents the fraction of cells contributing to the clusters with higher resolution, the color of the arrows shows the number of cells that move from one cluster to another. The letters on the left of each level univocally identify the sub clusters of each cell type (as in Fig. 2A). (**C**) Dot plot showing the top 15 DEGs identified between the two sub clusters of the PE (d5 +RA) (PE(d5 +RA)_D3 and PE(d5 +RA)_D8; bottom level in (B)) and their expression levels (log[norm.counts+1]) in all the subclusters. The dot size represents the percentage of cluster cells expressing the gene and the color represents the average expression in the cluster. (**D**) Representative immunofluorescence staining of known PE and DE markers performed in: hESCs, DE (d2) AFE (d5 -RA) and PE (d5 +RA). In Blue the DAPI and in Green the respective marker. Scale bar = 50um (**E**) Histograms showing the mRUBY expression (mCherry filter) in cells at different differentiation timepoint in H9-WT (negative control) and TBX1flag-mRUBY.
