# Supplementary material for: Transcriptional and epigenetic characterization of a new in vitro platform to model the formation of human pharyngeal endoderm
Source: Genome Biol. 2024 Aug 8;25:211. doi: 10.1186/s13059-024-03354-z (PMC11312149; doi:10.1186/s13059-024-03354-z)
Supplement: Supplementary file 6 — Additional file 6. Supplementary figure S3. [file 13059_2024_3354_MOESM6_ESM.pdf]

Figure S3

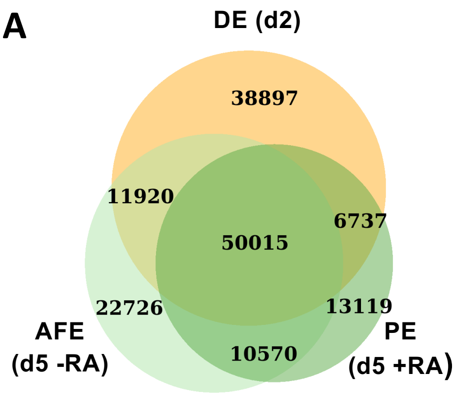

	Lose	Gain
DE vs AFE	41252	25553
DE vs PE	39146	19694
AFE vs PE	20907	14870

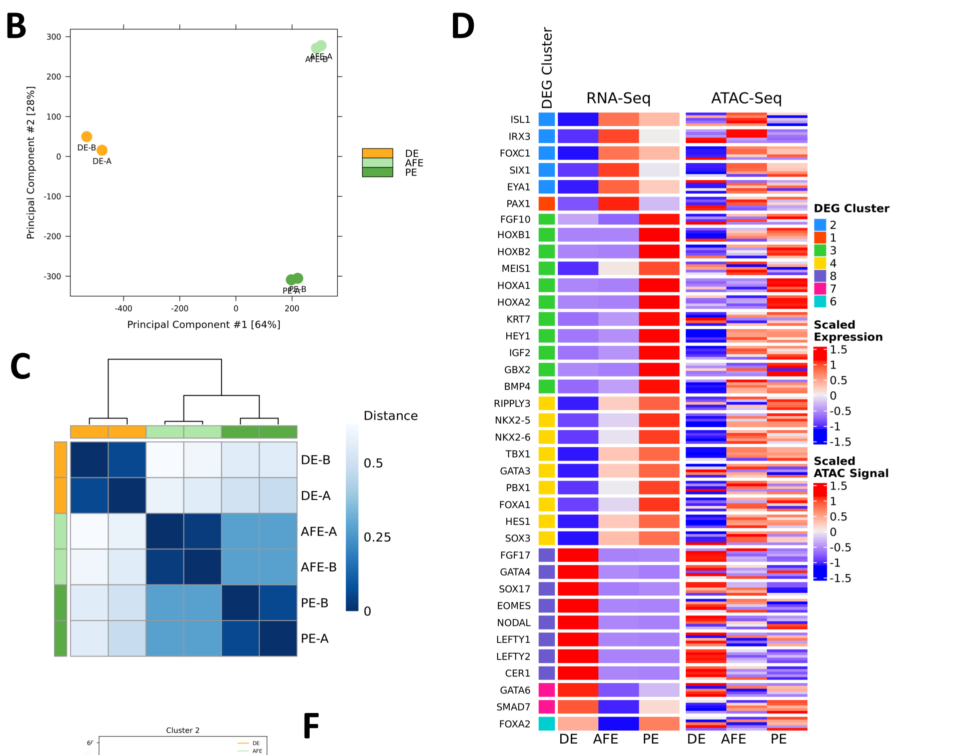

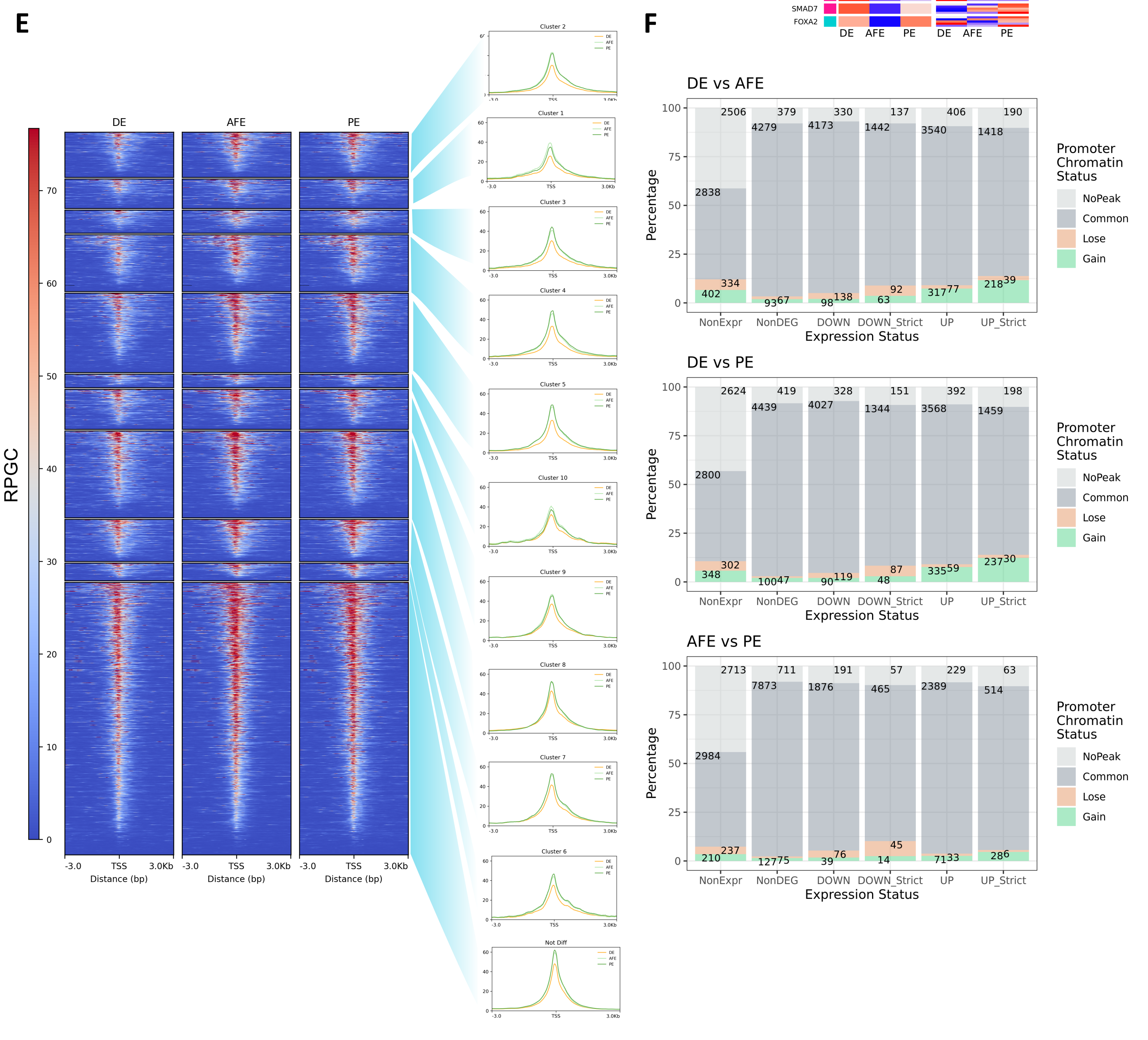

G

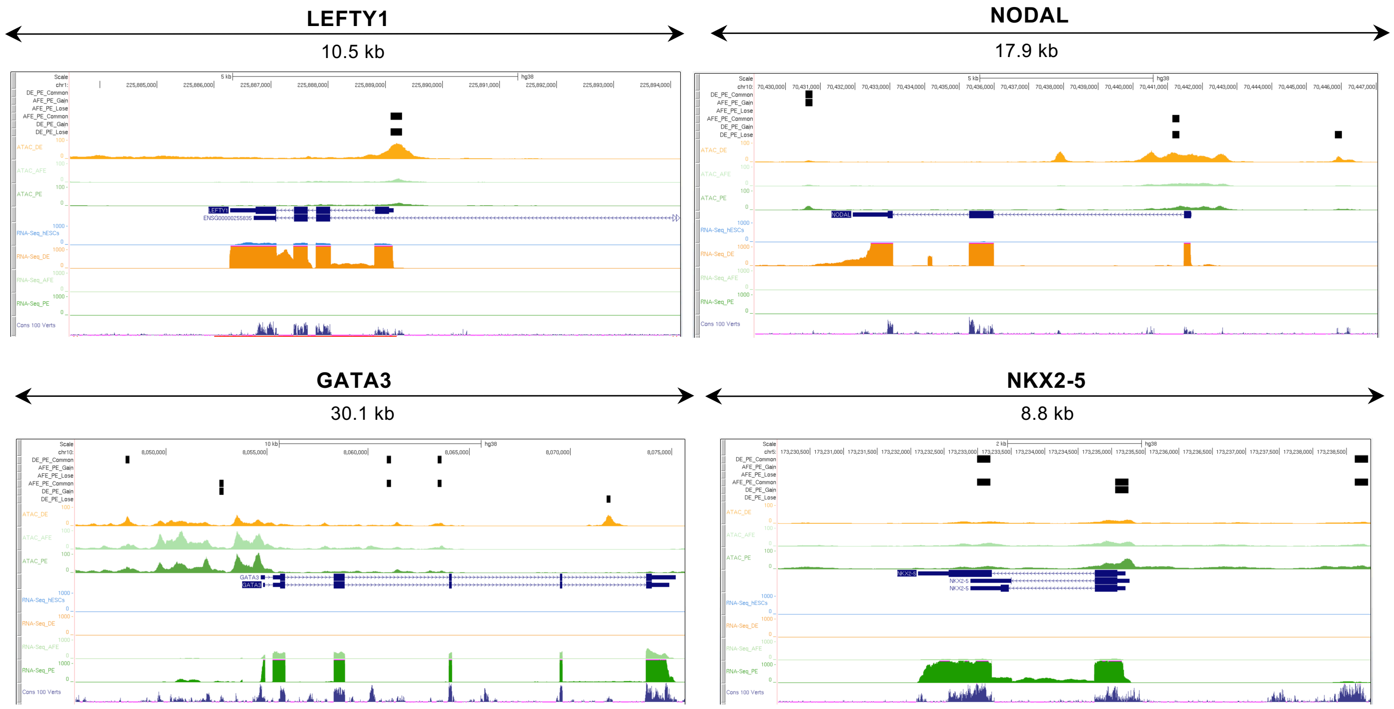
LEFTY1
10.5 kb
NODAL
17.9 kb
GATA3
30.1 kb
NKX2-5
8.8 kb

H

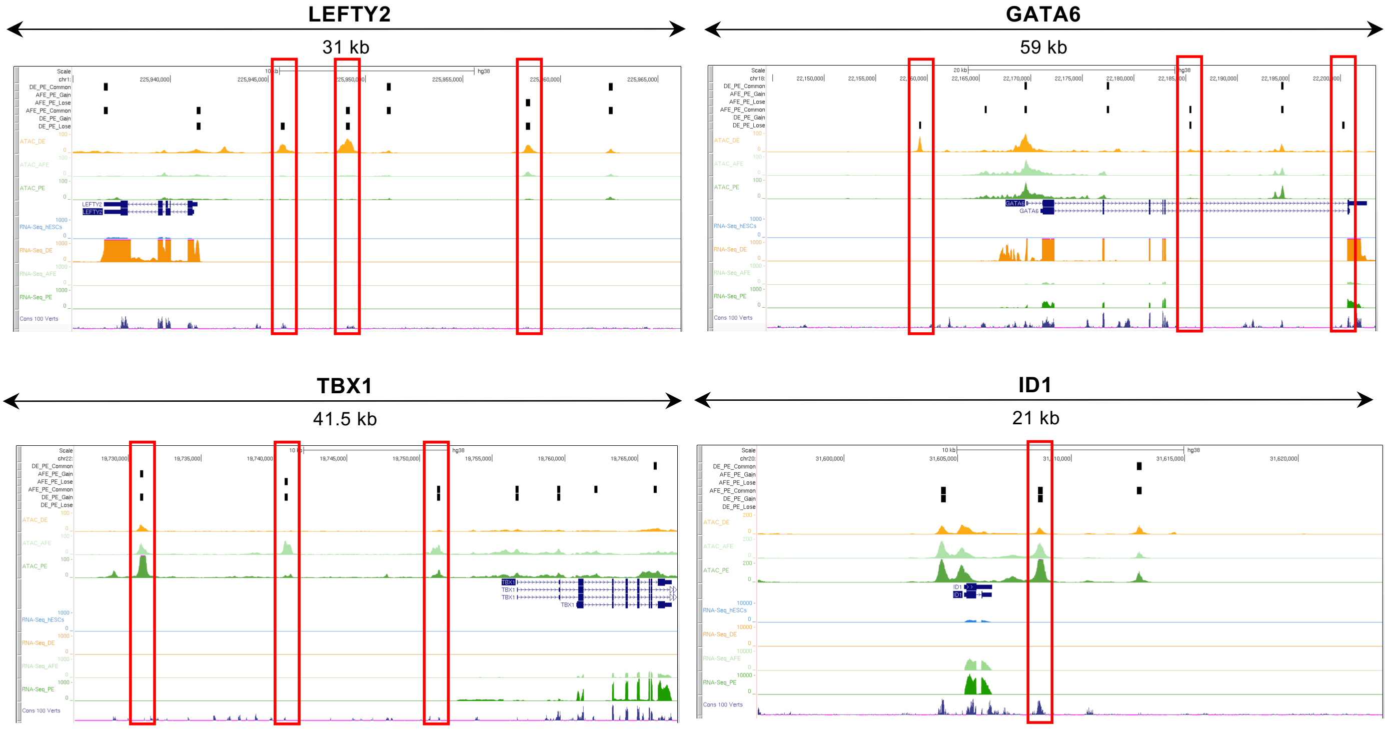
LEFTY2
31 kb
GATA6
59 kb
TBX1
41.5 kb
ID1
21 kb

Figure S3: ATAC-Seq analysis of DE (d2), AFE (d5 -RA), and PE (d5 +RA) cell types (related to Fig. 3).
(A) Venn diagram showing the consensus ATAC-Seq peak sets identified in each condition and their overlaps (upper panel). Table showing, for each differential accessibility contrast, the number of Gain (absolute log2[FC] > 1, FDR < 0.01) and Lose (absolute log2[FC] < -1, FDR < 0.01) peaks; see also Additional file 7. **(B)** PCA plot showing the clustering of DE (d2), AFE (d5 -RA), and PE (d5 +RA) samples based on their chromatin accessibility profile measured via ATAC-Seq of biological duplicates. Principal component 1 (PC1) and 2 (PC2) were identified based on the normalized read counts calculated for all peaks. **(C)** Sample-to-sample Pearson correlation heatmap showing the similarity of DE (d2), AFE (d5 -RA), and PE (d5 +RA) based on their chromatin accessibility profile measured *via* ATAC-Seq of biological duplicates. Sample-to-sample distances were calculated as 1-Pearson correlation of the normalized read counts computed for all peaks. Such distances were also used to draw the dendrogram showing the hierarchical clustering of samples. **(D)** Heatmaps showing, for each gene marker reported in Fig. 1C, the average gene expression (left heatmap) and the mean accessibility of the 5 ATAC-Seq consensus peaks closest to their respective transcription start sites (right heatmap, for each gene peaks are ordered by increasing distance), measured in the DE (d2), AFE (d5 -RA), and PE (d5 +RA) conditions. The expression values reported in the left heatmap correspond to row-scaled (Z-score), average rlog-transformed count data. The ATAC-Seq signal values reported in the right heatmap correspond to row-scaled (Z-score) average log2-transformed library size-normalized count data. **(E)** Heatmaps showing DE (d2), AFE (d5 -RA), and PE (d5 +RA) ATAC-Seq signal in 6 kb-long regions around the TSSs of protein-coding non-DEGs and DEGs belonging to the clusters shown in Fig. 1C. Signal was calculated on merged replicates as Reads Per Genome Coverage (RPGC) values with a bin size of 50 bp. Summary plots reporting the position-specific average signal calculated for each cluster are shown on the right. **(F)** Bar plots showing the promoter chromatin accessibility status (no ATAC-Seq peak, presence of Common, Gain or Lose peak) for non-expressed genes, non-DEGs and DEGs (all protein-coding) identified in each contrast. Promoters were defined as TSS ± 3 kb. NoPeak: no ATAC-Seq peak was found in any of the gene promoters; Common: at least one Common peak was found; Lose: at least one Lose peak was found; Gain: at least one Gain peak was found; NonExpr: average TPM < 1 in both the conditions of the contrast; NonDEG: the gene is not differentially expressed; DOWN: the gene is downregulated (log2[FC] significantly < 0); DOWN_Strict: the gene is strongly downregulated (log2[FC] significantly < -0.58); UP: the gene is upregulated (log2[FC] significantly > 0); UP_Strict: the gene is strongly upregulated (log2[FC] significantly > 0.58). **(G)** Visualization of genomic regions encompassing known DE (top panel) and PE (d5 +RA) (bottom panel) marker gene loci whose promoter hosts a DAR that, respectively, decreases or increases its accessibility during the differentiation to PE (d5 +RA), along with tracks relative to (from top to bottom): Bulk RNA-Seq coverage (FPKM values of pooled hESCs, DE (d2), AFE (d5 -RA), and PE (d5 +RA) replicates), GENCODE transcripts, ATAC-Seq coverage (RPGC values of pooled DE (d2), AFE (d5 -RA), and PE (d5 +RA) replicates), ATAC-Seq peaks (classified based on the AFE (d5 -RA) vs PE (d5 +RA) and DE (d2) vs PE (d5 +RA) contrasts) and Vertebrate PhyloP conservation. The image was produced using the UCSC Genome Browser. **(H)** Visualization of genomic regions encompassing known DE (d2) (top panel) and PE (d5 +RA) (bottom panel) marker gene loci close to non-promoter DARs that, respectively, decrease or increase their accessibility during the differentiation to PE (d5 +RA) (highlighted by red boxes), along with tracks relative to (from top to bottom): Bulk RNA-Seq coverage (FPKM values of pooled hESCs, DE (d2), AFE (d5 -RA), and PE (d5 +RA) replicates), GENCODE transcripts, ATAC-Seq coverage (RPGC values of pooled DE (d2), AFE (d5 -RA), and PE (d5 +RA) replicates), ATAC-Seq peaks (classified based on the AFE (d5 -RA) vs PE (d5 +RA) and DE (d2) vs PE (d5 +RA) contrasts) and Vertebrate PhyloP conservation. The image was produced using the UCSC Genome Browser.
