# Supplementary material for: Transcriptional and epigenetic characterization of a new in vitro platform to model the formation of human pharyngeal endoderm
Source: Genome Biol. 2024 Aug 8;25:211. doi: 10.1186/s13059-024-03354-z (PMC11312149; doi:10.1186/s13059-024-03354-z)
Supplement: Supplementary file 9 — Additional file 9. Supplementary figure S4. [file 13059_2024_3354_MOESM9_ESM.pdf]

Figure S4

A

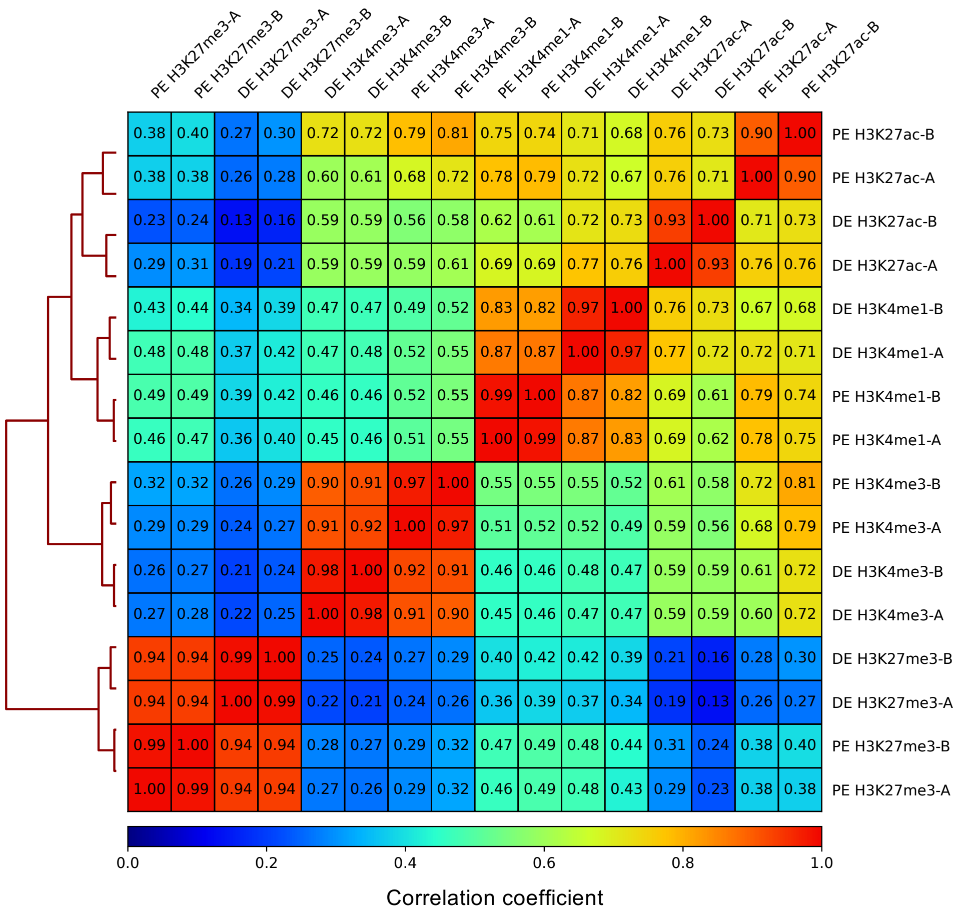

Promoters

B

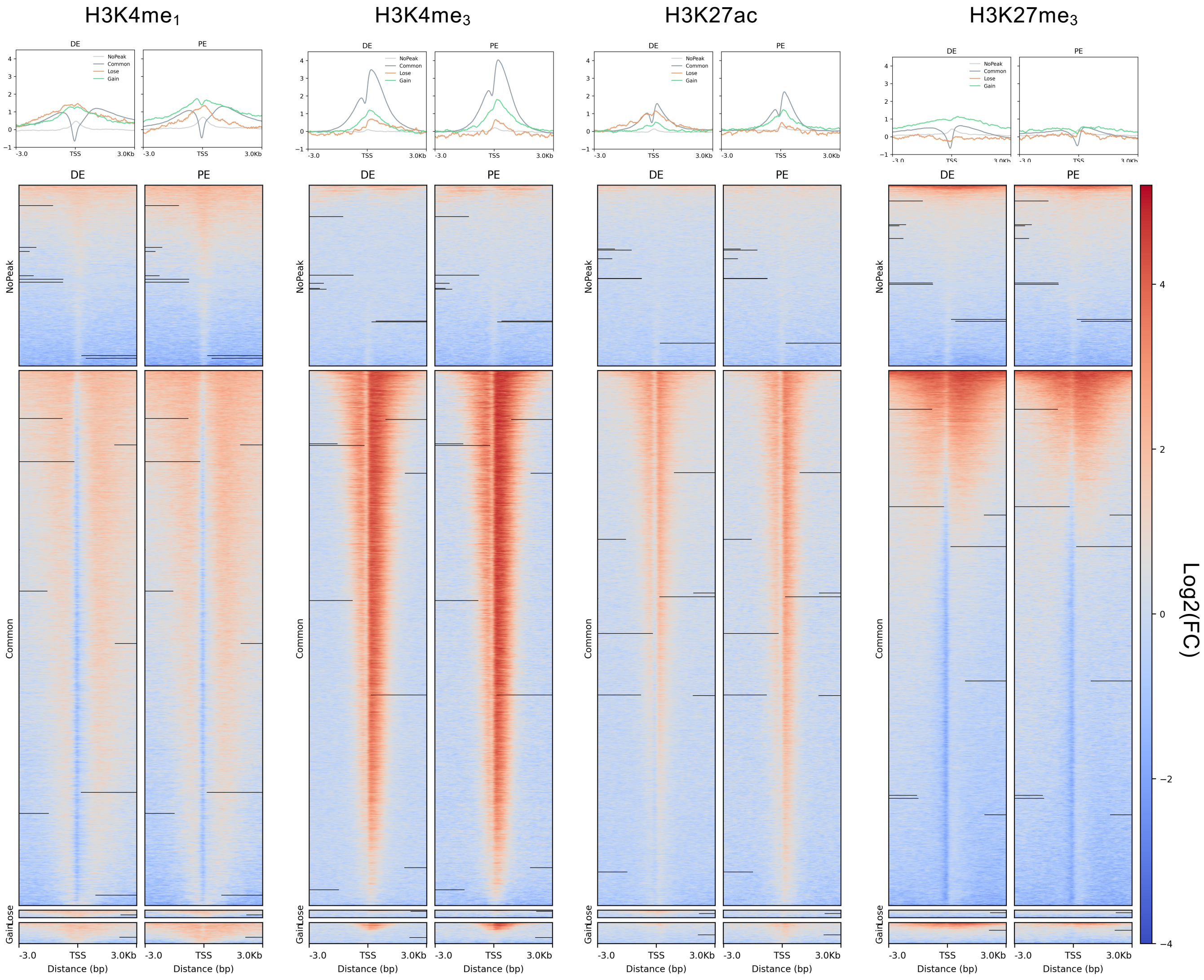

C

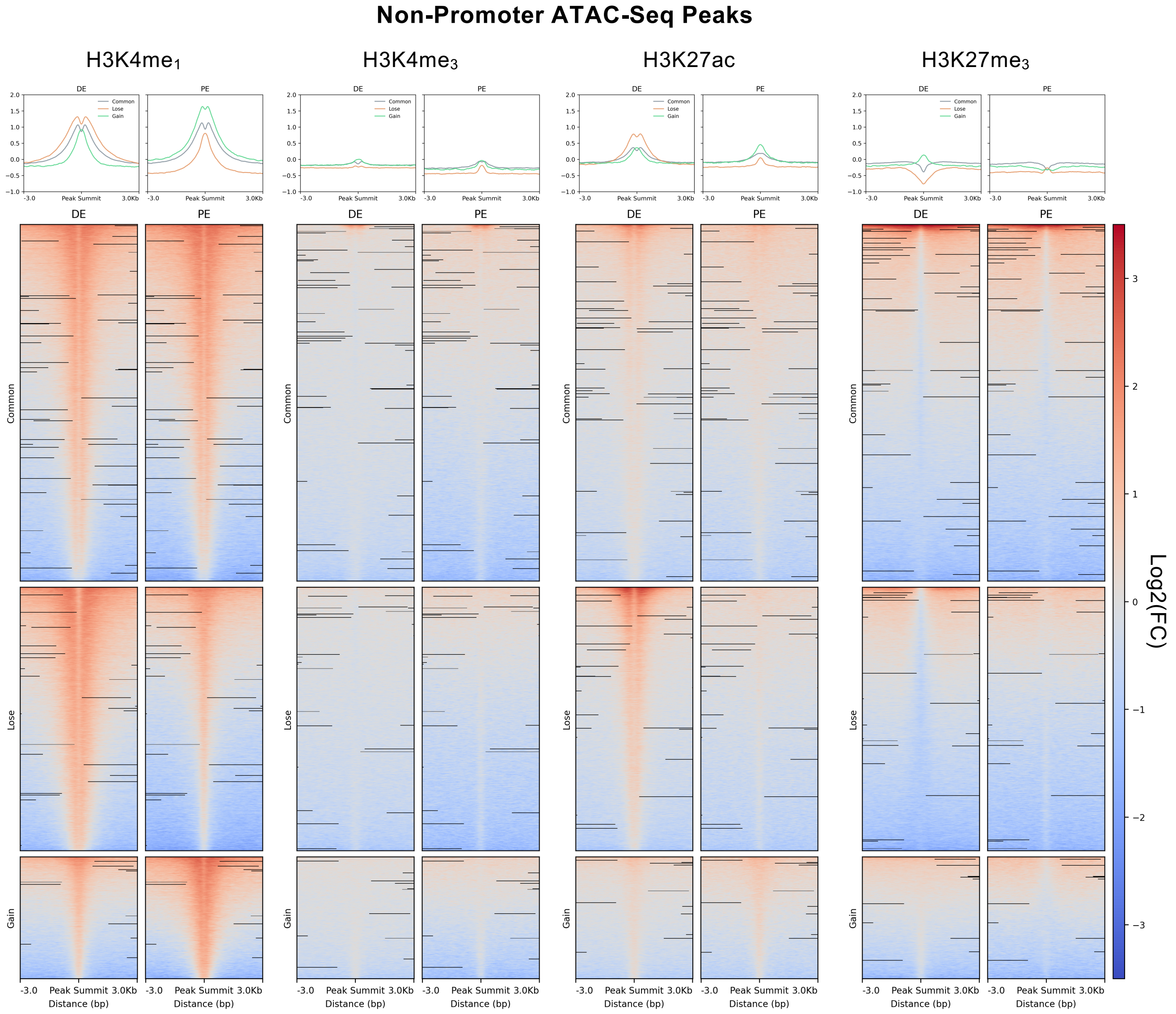
Non-Promoter ATAC-Seq Peaks
H3K4me1
H3K4me3
H3K27ac
H3K27me3
DE
PE
Common
Lose
Gain
Peak Summit
Distance (bp)
-3.0
3.0Kb
Log2(FC)

D

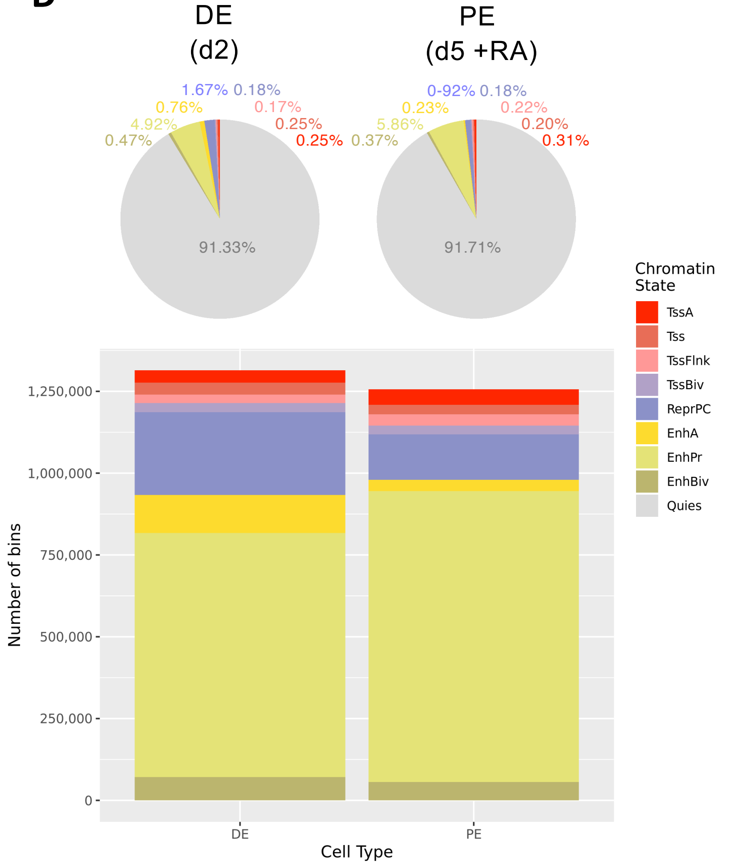
DE
(d2)
PE
(d5 +RA)
1.67% 0.18%
0.76%
0.17%
4.92%
0.25%
0.47%
0.25%
91.33%
0-92% 0.18%
0.23%
0.22%
5.86%
0.20%
0.37%
0.31%
91.71%
Chromatin State
TssA
Tss
TssFlnk
TssBiv
ReprPC
EnhA
EnhPr
EnhBiv
Quies
Number of bins
1,250,000
1,000,000
750,000
500,000
250,000
0
DE
PE
Cell Type

E

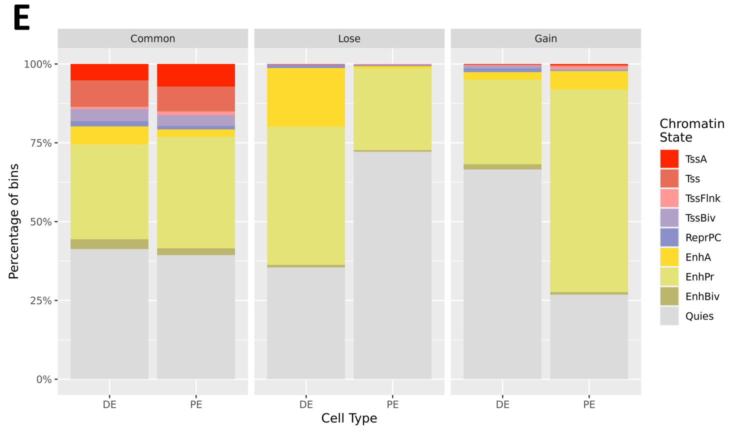
Common
Lose
Gain
Percentage of bins
100%
75%
50%
25%
0%
DE
PE
Cell Type
Chromatin State
TssA
Tss
TssFlnk
TssBiv
ReprPC
EnhA
EnhPr
EnhBiv
Quies

F

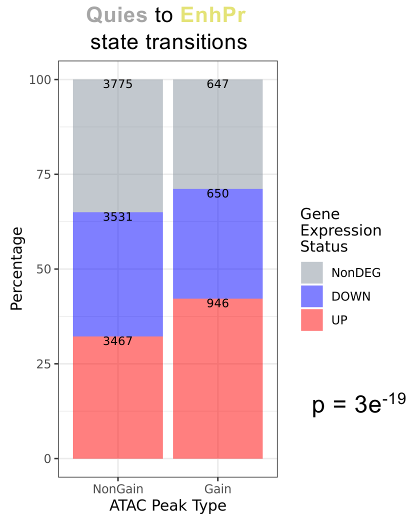
Quies to EnhPr
state transitions
Percentage
100
75
50
25
0
3775
3531
3467
647
650
946
NonGain
Gain
ATAC Peak Type
Gene Expression Status
NonDEG
DOWN
UP
p = 3e-19

G

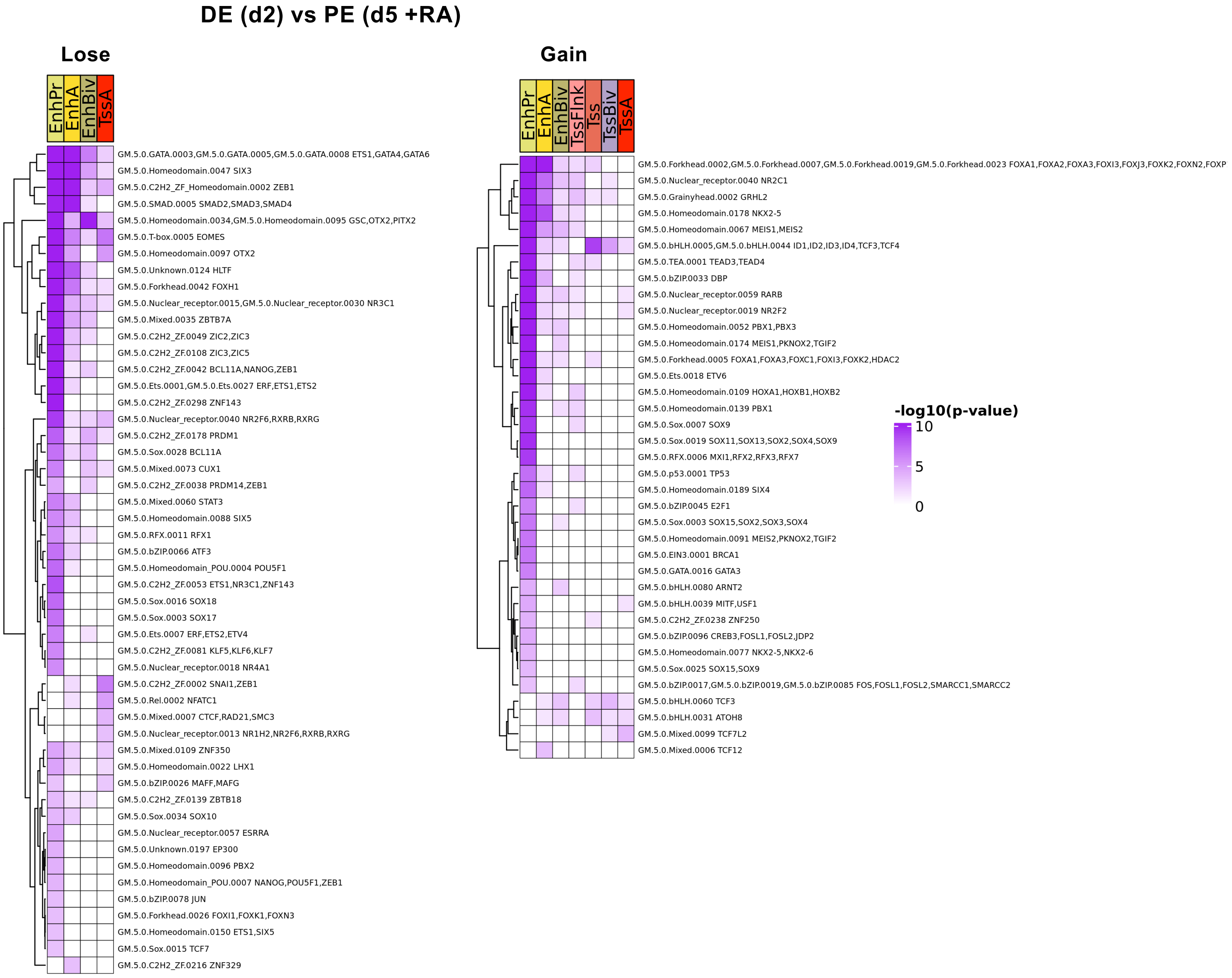

DE (d2) vs PE (d5 +RA)
Lose
EnhPr
EnhA
EnhBiv
TssA
GM.5.0.GATA.0003,GM.5.0.GATA.0005,GM.5.0.GATA.0008 ETS1,GATA4,GATA6
GM.5.0.Homeodomain.0047 SIX3
GM.5.0.C2H2_ZF_Homeodomain.0002 ZEB1
GM.5.0.SMAD.0005 SMAD2,SMAD3,SMAD4
GM.5.0.Homeodomain.0034,GM.5.0.Homeodomain.0095 GSC,OTX2,PITX2
GM.5.0.T-box.0005 EOMES
GM.5.0.Homeodomain.0097 OTX2
GM.5.0.Unknown.0124 HLTF
GM.5.0.Forkhead.0042 FOXH1
GM.5.0.Nuclear_receptor.0015,GM.5.0.Nuclear_receptor.0030 NR3C1
GM.5.0.Mixed.0035 ZBTB7A
GM.5.0.C2H2_ZF.0049 ZIC2,ZIC3
GM.5.0.C2H2_ZF.0108 ZIC3,ZIC5
GM.5.0.C2H2_ZF.0042 BCL11A,NANOG,ZEB1
GM.5.0.Ets.0001,GM.5.0.Ets.0027 ERF,ETS1,ETS2
GM.5.0.C2H2_ZF.0298 ZNF143
GM.5.0.Nuclear_receptor.0040 NR2F6,RXRB,RXRG
GM.5.0.C2H2_ZF.0178 PRDM1
GM.5.0.Sox.0028 BCL11A
GM.5.0.Mixed.0073 CUX1
GM.5.0.C2H2_ZF.0038 PRDM14,ZEB1
GM.5.0.Mixed.0060 STAT3
GM.5.0.Homeodomain.0088 SIX5
GM.5.0.RFX.0011 RFX1
GM.5.0.bZIP.0066 ATF3
GM.5.0.Homeodomain_POU.0004 POU5F1
GM.5.0.C2H2_ZF.0053 ETS1,NR3C1,ZNF143
GM.5.0.Sox.0016 SOX18
GM.5.0.Sox.0003 SOX17
GM.5.0.Ets.0007 ERF,ETS2,ETV4
GM.5.0.C2H2_ZF.0081 KLF5,KLF6,KLF7
GM.5.0.Nuclear_receptor.0018 NR4A1
GM.5.0.C2H2_ZF.0002 SNAI1,ZEB1
GM.5.0.Rel.0002 NFATC1
GM.5.0.Mixed.0007 CTCF,RAD21,SMC3
GM.5.0.Nuclear_receptor.0013 NR1H2,NR2F6,RXRB,RXRG
GM.5.0.Mixed.0109 ZNF350
GM.5.0.Homeodomain.0022 LHX1
GM.5.0.bZIP.0026 MAFF,MAFG
GM.5.0.C2H2_ZF.0139 ZBTB18
GM.5.0.Sox.0034 SOX10
GM.5.0.Nuclear_receptor.0057 ESRRA
GM.5.0.Unknown.0197 EP300
GM.5.0.Homeodomain.0096 PBX2
GM.5.0.Homeodomain_POU.0007 NANOG,POU5F1,ZEB1
GM.5.0.bZIP.0078 JUN
GM.5.0.Forkhead.0026 FOXI1,FOXK1,FOXN3
GM.5.0.Homeodomain.0150 ETS1,SIX5
GM.5.0.Sox.0015 TCF7
GM.5.0.C2H2_ZF.0216 ZNF329
Gain
EnhPr
EnhA
EnhBiv
TssFlnk
Tss
TssBiv
TssA
GM.5.0.Forkhead.0002,GM.5.0.Forkhead.0007,GM.5.0.Forkhead.0019,GM.5.0.Forkhead.0023 FOXA1,FOXA2,FOXA3,FOXI3,FOXJ3,FOXK2,FOXN2,FOXP
GM.5.0.Nuclear_receptor.0040 NR2C1
GM.5.0.Grainyhead.0002 GRHL2
GM.5.0.Homeodomain.0178 NKX2-5
GM.5.0.Homeodomain.0067 MEIS1,MEIS2
GM.5.0.bHLH.0005,GM.5.0.bHLH.0044 ID1,ID2,ID3,ID4,TCF3,TCF4
GM.5.0.TEA.0001 TEAD3,TEAD4
GM.5.0.bZIP.0033 DBP
GM.5.0.Nuclear_receptor.0059 RARB
GM.5.0.Nuclear_receptor.0019 NR2F2
GM.5.0.Homeodomain.0052 PBX1,PBX3
GM.5.0.Homeodomain.0174 MEIS1,PKNOX2,TGIF2
GM.5.0.Forkhead.0005 FOXA1,FOXA3,FOXC1,FOXI3,FOXK2,HDAC2
GM.5.0.Ets.0018 ETV6
GM.5.0.Homeodomain.0109 HOXA1,HOXB1,HOXB2
GM.5.0.Homeodomain.0139 PBX1
GM.5.0.Sox.0007 SOX9
GM.5.0.Sox.0019 SOX11,SOX13,SOX2,SOX4,SOX9
GM.5.0.RFX.0006 MXI1,RFX2,RFX3,RFX7
GM.5.0.p53.0001 TP53
GM.5.0.Homeodomain.0189 SIX4
GM.5.0.bZIP.0045 E2F1
GM.5.0.Sox.0003 SOX15,SOX2,SOX3,SOX4
GM.5.0.Homeodomain.0091 MEIS2,PKNOX2,TGIF2
GM.5.0.EIN3.0001 BRCA1
GM.5.0.GATA.0016 GATA3
GM.5.0.bHLH.0080 ARNT2
GM.5.0.bHLH.0039 MITF,USF1
GM.5.0.C2H2_ZF.0238 ZNF250
GM.5.0.bZIP.0096 CREB3,FOSL1,FOSL2,JDP2
GM.5.0.Homeodomain.0077 NKX2-5,NKX2-6
GM.5.0.Sox.0025 SOX15,SOX9
GM.5.0.bZIP.0017,GM.5.0.bZIP.0019,GM.5.0.bZIP.0085 FOS,FOSL1,FOSL2,SMARCC1,SMARCC2
GM.5.0.bHLH.0060 TCF3
GM.5.0.bHLH.0031 ATOH8
GM.5.0.Mixed.0099 TCF7L2
GM.5.0.Mixed.0006 TCF12
-log10(p-value)
10
5
0

Figure S4: Histone modification ChIP-Seq analysis of DE (d2) and PE (d5 +RA) cell types (related to Fig. 5).

(A) Sample-to-sample Pearson correlation heatmap showing the similarity of DE (d2) and PE (d5 +RA) samples based on their H3K4me3, H3K27me3, H3K4me1, and H3K27ac Histone Mark profiles measured *via* ChIP-Seq of biological duplicates. Sample-to-sample distances were calculated as 1-Pearson correlation of mean Reads Per Genome Coverage (RPGC) scores computed over 10 kb-long genomic bins. Such distances were also used to draw the dendrogram showing the hierarchical clustering of samples. **(B)** Heatmaps showing DE (d2) and PE (d5 +RA) Histone Mark CHIP-Seq signal in 6 kb-long regions around the TSSs of protein-coding genes, stratified based on the overlap of the TSSs with Common ATAC-Seq peaks or DARs. Signal was calculated on merged replicates as log2-transformed fold change of the RPGC values over the input samples, with a bin size of 50 bp. Summary plots reporting the position-specific average signal calculated for each TSS class are shown above the heatmaps. **(C)** Heatmaps showing DE (d2) and PE (d5 +RA) Histone Mark CHIP-Seq signal in 6 kb-long regions around the summits of ATAC-Seq peaks localized outside promoter regions (TSS ± 3 kb), stratified based on the differential accessibility between DE (d2) and PE (d5 +RA). Signal was calculated on merged replicates as log2-transformed fold change of the RPGC values over the input samples, with a bin size of 50 bp. Summary plots reporting the position-specific average signal calculated for each TSS class are shown above the heatmaps. **(D)** Pie charts (upper panel) and bar plot (bottom panel) showing, respectively, the percentages and the counts of 200 bp-long genomic bins occupied by the different ChromHMM state calls in DE (d2) and PE (d5 +RA) cells. Quiescent states are not represented in the bar plot to better show the differences in the coverage of the other states. **(E)** Bar plot showing the chromatin state composition in DE (d2) and PE (d5 +RA) cells of 200 bp-long genomic bins that overlap with the Common, Lose and Gain ATAC-Seq peaks identified in the DE (d2) vs PE (d5 +RA) contrast. **(F)** Bar plot showing, for the DE (d2) vs PE (d5 +RA) contrast, the expression status of the expressed protein-coding genes closest to the genomic regions transitioning from a Quies state in DE (d2) to an EnhPR state in PE (d5 +RA), stratified based on the overlap with Gain ATAC-Seq peaks. Nearest genes were searched within a 50 kb range. The reported p-value was calculated by comparing the proportions of upregulated genes between the two classes of genes *via* Fisher's exact test. NonDEG: the gene is not differentially expressed; DOWN: the gene is downregulated (log2[FC] significantly < 0); UP: the gene is upregulated (log2[FC] significantly > 0); NonGain: the genomic region does not overlap with a Gain peak summit; Gain: the genomic region overlaps with a Gain peak summit. (**G**) Heatmaps showing TF FPs enriched in DE (d2) vs PE (d5 +RA) Lose (left panel) and Gain (right panel) DARs, stratified based on their DE (d2) and PE (d5 +RA) chromatin state, respectively. For the analysis of Lose DARs, only transitions enriched in Lose peaks and downregulated TFs with an average DE (d2) TPM > 5 were used; for the analysis of Gain DARs, only transitions enriched in Gain peaks and upregulated TFs with an average PE (d5 +RA) TPM > 5 were employed. Each row refers to one or more GimmeMotifs vertebrate motifs having identical enrichment profile and the corresponding TFs. Redundant motifs were excluded for better visualization. Cell color intensities are proportional to the –log10 transformed BiFET p-value; only motifs having p-value < 0.001 in at least one DAR class are reported.
