# Supplementary material for: Transcriptional and epigenetic characterization of a new in vitro platform to model the formation of human pharyngeal endoderm
Source: Genome Biol. 2024 Aug 8;25:211. doi: 10.1186/s13059-024-03354-z (PMC11312149; doi:10.1186/s13059-024-03354-z)
Supplement: Supplementary file 11 — Additional file 11. Supplementary figure S5. [file 13059_2024_3354_MOESM11_ESM.pdf]

Figure S5

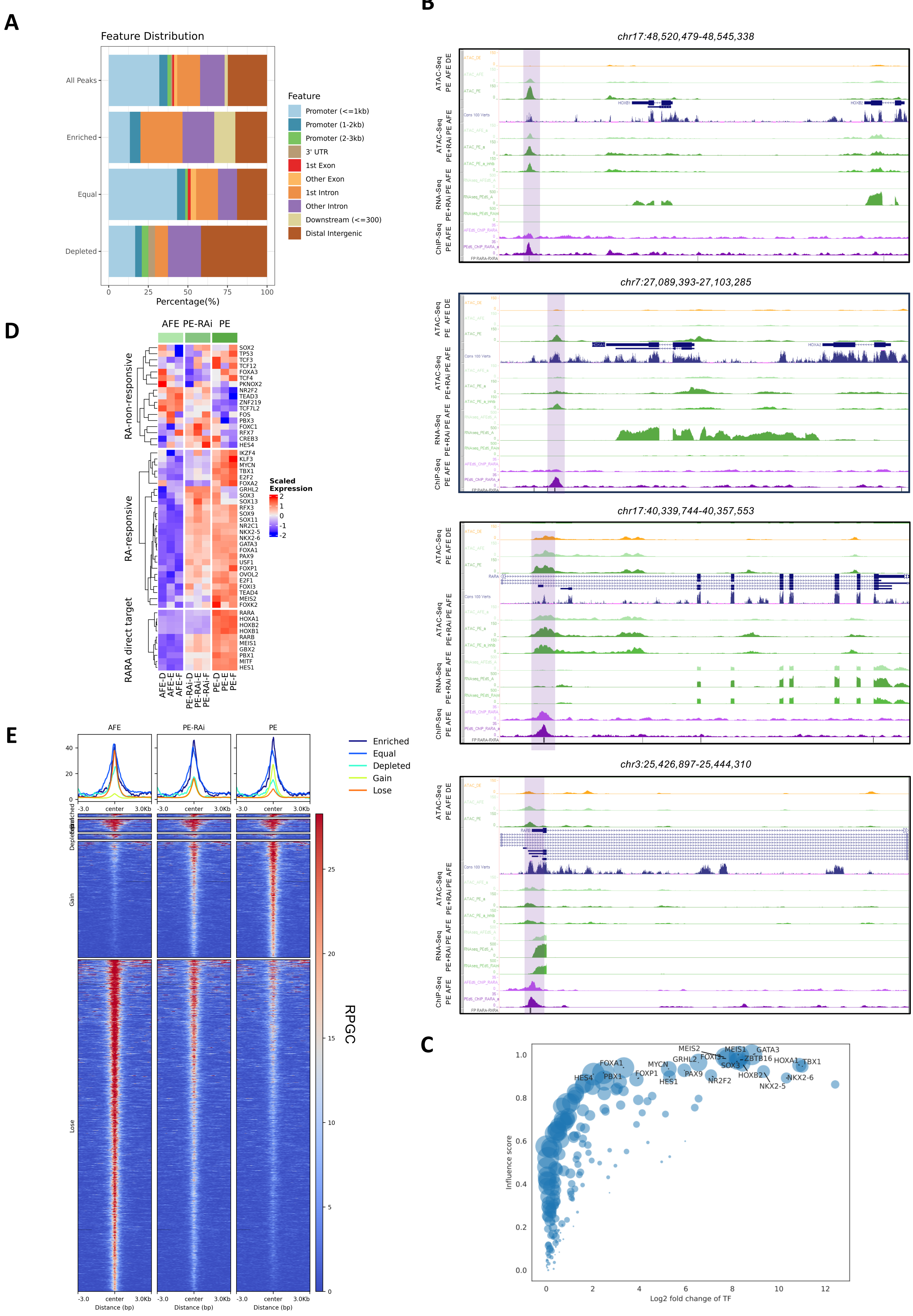

A
Feature Distribution
All Peaks
Enriched
Equal
Depleted
0
25
50
75
100
Percentage(%)
Feature
Promoter (<=1kb)
Promoter (1-2kb)
Promoter (2-3kb)
3' UTR
1st Exon
Other Exon
1st Intron
Other Intron
Downstream (<=300)
Distal Intergenic
B
chr17:48,520,479-48,545,338
chr7:27,089,393-27,103,285
chr17:40,339,744-40,357,553
chr3:25,426,897-25,444,310
ATAC-Seq PE AFE DE
ATAC-Seq PE+RAi PE AFE
RNA-Seq PE+RAi PE AFE
ChIP-Seq PE AFE
HOXB1
HOXB2
HOXA1
HOXA2
RARA
RARB
C
Influence score
Log2 fold change of TF
MEIS2
GRHL2
FOXI3
MEIS1
GATA3
ZBTB16
HOXA1
TBX1
FOXA1
MYCN
SOX3
HES4
PBX1
FOXP1
PAX9
NR2F2
HOXB2
NKX2-6
HES1
NKX2-5
D
AFE
PE-RAi
PE
RA-non-responsive
RA-responsive
RARA direct target
SOX2
TP53
TCF3
TCF12
FOXA3
TCF4
PKNOX2
NR2F2
TEAD3
ZNF219
TCF7L2
FOS
PBX3
FOXC1
RFX7
CREB3
HES4
IKZF4
KLF3
MYCN
TBX1
E2F2
FOXA2
GRHL2
SOX3
SOX13
RFX3
SOX9
SOX11
NR2C1
NKX2-5
NKX2-6
GATA3
FOXA1
PAX9
USF1
FOXP1
OVOL2
E2F1
FOXI3
TEAD4
MEIS2
FOXK2
RARA
HOXA1
HOXB2
HOXB1
RARB
MEIS1
GBX2
PBX1
MITF
HES1
Scaled Expression
2
1
0
-1
-2
AFE-D
AFE-E
AFE-F
PE-RAi-D
PE-RAi-E
PE-RAi-F
PE-D
PE-E
PE-F
E
AFE
PE-RAi
PE
Enriched
Equal
Depleted
Gain
Lose
Gain
Lose
-3.0
center
3.0Kb
Distance (bp)
RPGC

Figure S5: Integration of RNA-Seq, ATAC-Seq, and ChIP-Seq data (related to Fig. 6).
(A) Bar plot illustrating the genomic annotation distribution of all RARA ChIP-Seq peaks, as well as those categorized as Enriched, Equal, or Depleted. Each genomic feature is represented by a specific color shown in the legend. (**B**) UCSC genome browser visualization of genomic regions encompassing the HOXA, HOXB, RARA, and RARB gene loci, and along with tracks relative to (from top to bottom): ATAC-Seq coverage of DE (d2), AFE (d5 -RA), and PE (d5 +RA) from Differentiation_1, GENCODE transcripts, Vertebrate PhyloP conservation, ATAC-Seq coverage of AFE (d5 -RA), PE (d5 +RA), and PE-RAi (d5 +RA +AGN193109) from Differentiation_2 (see Methods), Bulk RNA-Seq coverage of AFE (d5 -RA), PE (d5 +RA), and PE-RAi (d5 +RA) from Differentiation_2, RARA ChIP-Seq coverage of AFE (d5 -RA) and PE (d5 +RA), and RARE FPs identified in PE. Purple boxes indicate the position of the RARA ChIP-Seq peaks. **(C)** Bubble plot showing the Ananse TF sumScaled influence scores for the transition from DE (d2) to PE (d5 +RA), along with the log2(FC) calculated for the DE (d2) vs PE (d5 +RA) contrast. Bubble size is proportional to the number of direct targets. The top 20 TFs based on influence score are labelled with their gene name. (**D**) Heatmap showing the expression in AFE (d5 -RA), PE (d5 +RA), and PE-RAi (d5 +RA +AGN193109) samples of the TFs belonging to the TRN, divided in the 3 functional groups shown in B. Hierarchical clustering of the three groups also shown; see also The expression values reported in the heatmap correspond to row-scaled (Z-score), rlog-transformed count data. (**E**) Heatmaps showing the ATAC-Seq signal, measured in AFE (d5 -RA), PE-RAi (d5 +RA +AGN193109), and PE (d5 +RA) samples from Differentiation_2 experiment (see Methods), within 6 kb-long regions centered around the summits of Enriched, Equal, and Depleted RARA ChIP-Seq peaks, and of ATAC-Seq Gain and Lose peaks identified by comparing Differentiation_2 AFE (d5 -RA) and PE (d5 +RA) samples. ATAC-Seq Signal was calculated on merged replicates as RPGC values with a bin size of 50 bp. Summary plots reporting the position-specific average signal calculated for each cell type and peak category are shown on top.
