# Supplementary material for: Transcriptional and epigenetic characterization of a new in vitro platform to model the formation of human pharyngeal endoderm
Source: Genome Biol. 2024 Aug 8;25:211. doi: 10.1186/s13059-024-03354-z (PMC11312149; doi:10.1186/s13059-024-03354-z)
Supplement: Supplementary file 14 — Additional file 14. Review history. [file 13059_2024_3354_MOESM14_ESM.docx]

Review History

**First round of review**

**Reviewer 1**

**Were you able to assess all statistics in the manuscript, including the appropriateness of statistical tests used?**

Yes, and I have assessed the statistics in my report.

**Comments to author:**

The manuscript presents a comprehensive study on the transcriptional and epigenetic characterisation of human Pharyngeal Endoderm (PE)-like cells derived from human Embryonic Stem Cells (hESCs). The research fills a significant knowledge gap by providing an integrated multi-omics characterization of PE-like cells, demonstrating their transcriptional homogeneity and similarity to in vivo mouse PE cells. The study identifies new putative regulatory regions and constructs a Retinoic Acid-centered transcription factor network, offering insights into the epigenetic dynamics of human PE differentiation.

A few minor concerns for improvement include:

1. The part of the comparison with Mouse scRNA-Seq analysis is innovative as it seeks to compare the obtained human PE cell data with mouse single-cell RNA-Seq data to identify similarities in gene expression patterns across species. Such cross-species analyses are crucial for validating the relevance of model organisms in studying human biology. The comparisons could be enhanced by including functional annotations, gene set enrichment analyses, or similar analyses to better understand the biological significance of the observed similarities.

2. The authors have made significant strides in characterising PE cells; the authors should be careful in highlighting that, at the moment, this is primarily a descriptive genomics work, and further experiments aimed at demonstrating how these elements contribute to specific functional tissues are yet to be exploited.

**Reviewer 2**

**Were you able to assess all statistics in the manuscript, including the appropriateness of statistical tests used?**

Yes, and I have assessed the statistics in my report.

**Comments to author:**

 In this manuscript, the epigenetic dynamics of human PE differentiation are revealed by combining hESCs differentiation with computational genomics, providing a solid resource and foundation for the development of PE derivatives and their modeling of developmental defects in genetic syndromes.Overall, this paper is very interesting. However, there are other issues that need to be addressed before the paper is suitable for publication in genome biology.

Major points:

1. Fig1 : B. C involves a large number of marker genes for AFE or PE, but not all genes provide relevant references.

2. Fig 1D: There is not a clear definition of the vertical axis in the figure, and the phase system development is not the most significant.

3. Fig 1E: Why is the time different, for example, the clusters gathered in 9 days and 9.5 days are different? Can we gather in the same cluster with different values?

4. The Figures in the manuscript need to be polished, and the font size and format are very inconsistent, such as Figure 3.

5.In Figure 1, a large number of marker genes are mentioned. Can the mRNA expression and chromatin openness of these genes be jointly analyzed?

6. In Figure 4. Only analyzing the expression differences of transcription factors between cells did not describe the reasons for the differences and the possible consequences of these differences.

7. Figure 6 shows the modification effect of histone modifications on related TFs, but does not combine transcriptome information. It is expected that transcriptome, chromatin openness, and chip seq data can be jointly analyzed to demonstrate the importance of chromatin structure in cell differentiation.

8. This manuscript makes a lot of inferences, but lacks some reliable validation experiments.

Minor points:

1. Please provide the model and size of the labels font in the manuscript, as it is too cluttered.

2. There are still some language issues, please find the relevant mother tongue .

**Authors Response**

**Point-by-point responses to the reviewers’ comments:**

**Reviewer #1**

The manuscript presents a comprehensive study on the transcriptional and epigenetic characterisation of human Pharyngeal Endoderm (PE)-like cells derived from human Embryonic Stem Cells (hESCs). The research fills a significant knowledge gap by providing an integrated multi-omics characterization of PE-like cells, demonstrating their transcriptional homogeneity and similarity to in vivo mouse PE cells. The study identifies new putative regulatory regions and constructs a Retinoic Acid-centered transcription factor network, offering insights into the epigenetic dynamics of human PE differentiation.
A few minor concerns for improvement include:

**1. The part of the comparison with Mouse scRNA-Seq analysis is innovative as it seeks to compare the obtained human PE cell data with mouse single-cell RNA-Seq data to identify similarities in gene expression patterns across species. Such cross-species analyses are crucial for validating the relevance of model organisms in studying human biology. The comparisons could be enhanced by including functional annotations, gene set enrichment analyses, or similar analyses to better understand the biological significance of the observed similarities.**

*Reply: We thank the reviewer for appreciating our efforts to validate the quality of the in vitro-derived PE cells through cross-species analysis and for suggesting a strategy to corroborate our findings. To address this point, we have* *performed a GO BP term enrichment analysis on the gene markers of the e_b3, e_b4 and e_c3 murine cell types which are also notably expressed in the human PE condition. This analysis revealed an enrichment towards categories related to development, especially that of the pharyngeal system (most enriched category), further supporting the effectiveness of our PE differentiation protocol. The results of this analysis have been discussed in the main text (lines 217-223), illustrated with a new supplementary figure (****Supplementary Figure 1H****), and added in tabular form to* ***Additional File*** ***4****. The methodological details have been included in the “Methods” section (lines 737, 1073-1074).*

**2. The authors have made significant strides in characterizing PE cells; the authors should be careful in highlighting that, at the moment, this is primarily a descriptive genomics work, and further experiments aimed at demonstrating how these elements contribute to specific functional tissues are yet to be exploited.**

*Reply: We thank the reviewer for the valuable feedback on our manuscript. We appreciate the suggestion to clarify the scope of our study. As recommended, we have added a paragraph on the “Conclusions” section (lines 574-578). This paragraph acknowledges that our current findings are primarily descriptive and outlines our plans for future experimental work to explore the functional implications of the genomic elements identified in PE cells. We believe this addition adequately addresses their concerns and enriches the manuscript by clearly delineating the next steps for our research.*

**Reviewer #2**

In this manuscript, the epigenetic dynamics of human PE differentiation are revealed by combining hESCs differentiation with computational genomics, providing a solid resource and foundation for the development of PE derivatives and their modeling of developmental defects in genetic syndromes. Overall, this paper is very interesting. However, there are other issues that need to be addressed before the paper is suitable for publication in genome biology.

Major points:

**1. Fig1 : B. C involves a large number of marker genes for AFE or PE, but not all genes provide relevant references.**

*Reply: We thank the reviewer for the feedback. The marker genes we have used as evidence in Figure 1B and 1C have been used by several groups who study endoderm development, whose references are in the text. In order to address the reviewer’s request, we have now added a new sheet with the requested information to* ***Supplementary Table 2****.*

**2. Fig 1D: There is not a clear definition of the vertical axis in the figure, and the phase system development is not the most significant.**

*Reply: The rows of the heatmap correspond to the differentially expressed genes (DEG) clusters. We have added a title to the rows to clarify this. We acknowledge that the “Pharyngeal System Development” category is not the most enriched overall, however it is the GO BP term with the highest Enrichment Ratio for Cluster 4 (and not for Cluster 3, as we previously erroneously reported in the main text), which corresponds to genes whose expression at day 5 is boosted by the addition of Retinoic Acid. We apologize for the mistake, which we corrected by replacing cluster 3 with cluster 4 at line 187.*

**3.  Fig 1E: Why is the time different, for example, the clusters gathered in 9 days and 9.5 days are different? Can we gather in the same cluster with different values?**

*Reply:* ***Figure 1E*** *shows a comparison between the data we obtained from our bulk-RNA sequencing at different stages of our differentiation protocol (hESC, DE, AFE and PE, corresponding to the rows of the plot) and the cell clusters (corresponding to the columns) that Han and colleagues identified from single-cell RNA sequencing data obtained from different endodermal development stages (8.5, 9.0 and 9.5 days, each corresponding to a matrix, see* ***Supplementary Figure 1F*** *for clarification about cell clusters and their lineages). We have modified the “Results” section (lines 205-206) and the* ***Figure 1E*** *caption to better clarify this analysis. As mentioned in the figure caption and extensively explained in the “Methods” section, cell types were compared by calculating the Spearman correlation of the expression profiles relative to the top 10 transcription factor markers of each single-cell cluster. In order to properly understand the correlations between the two datasets, it is essential that the different time points are analyzed separately. By performing this analysis, we were able to ascertain that our PE cells are indeed similar to their mouse in vivo counterparts: e_b3 and e_b4 cell clusters from day 9.0 and e_c3 cell cluster from day 9.5.*

**4. The Figures in the manuscript need to be polished, and the font size and format are very inconsistent, such as Figure 3.**

*Reply: We thank the reviewer for their suggestions. We have adjusted all the figures where possible, so that the font and text size are now more consistent.*

**5. In Figure 1, a large number of marker genes are mentioned. Can the mRNA expression and chromatin openness of these genes be jointly analyzed?**

*Reply: We thank the reviewer for suggesting to further demonstrate the correlation between differential gene expression and differential chromatin opening by looking at the mRNA expression and accessibility status of the marker genes shown in* ***Figure 1****. We present this information for some markers in Supplementary* ***Figures 3F*** *and* ***3G*** *(now* ***3G*** *and* ***3H****, respectively). We have now added a new* ***Supplementary Figure*** *(****3D****) illustrating the expression profile of all the marker genes, along with changes in chromatin opening at the 5 ATAC-Seq peaks closest to their* *transcription start sites. This analysis, which clearly shows that changes in gene expression are mirrored by variations in chromatin accessibility, is now described at lines 324-326.*

**6. In Figure 4. Only analyzing the expression differences of transcription factors between cells did not describe the reasons for the differences and the possible consequences of these differences.**

*Reply: We are grateful to the reviewer for raising this point. As detailed in the main text and in the caption,* ***Figure 4*** *shows the transcription factors that are differentially active in the DE, AFE and PE cell types. This activity was measured not only by looking at gene expression, but also by performing transcription factor motif and footprint enrichment analyses on the differentially accessible regions. In the caption and the “Methods” section we show that the transcription factors shown in the figure have expression profiles that correlate with motif-based activity, strongly supporting their role as drivers of the differences between the three cell types.*

**7. Figure 6 shows the modification effect of histone modifications on related TFs, but does not combine transcriptome information. It is expected that transcriptome, chromatin openness, and chip seq data can be jointly analyzed to demonstrate the importance of chromatin structure in cell differentiation.**

*Reply: We apologize if this did not come across as clear as needed, but our data analysis did integrate all the datasets, hence we respectfully disagree with the reviewer on this comment.*

*The results shown in the section “Transcription factor regulatory network inference classified TFs based on RA responsiveness and on RARA-mediated direct activation” were indeed obtained by integrating many of the high-throughput data we produced, including transcriptomic, ATAC-Seq and ChIP-Seq data, as it is clearly stated in the main text (“To this aim, we used the ANANSE tool, which exploits RNA-Seq, ATAC-Seq, H3K27ac ChIP-Seq, and known TF motifs to build a differential regulatory network between two different cell types”) and in the caption.*

***Figure 6A*** *shows the ChIP-Seq-derived binding sites for RARA, many of which are bound by the receptor even in absence of RA, along with the chromatin accessibility of those regions.*

***Figure 6B*** *shows a TF regulatory network obtained by integrating the aforementioned data with the RNA-Seq and the Histone Mark ChIP-Seq. We further integrated these results with transcriptomic and chromatin accessibility changes observed upon RA signal inhibition, as shown in* ***Figure 6C*** *and in* ***Supplementary Figure 5****.*

*Finally, as for the integration of Histone Mark ChIP-Seq data with transcriptome and chromatin accessibility information, this has been extensively accomplished in the “Epigenetic characterization of regulatory elements reveals functional chromatin state changes between DE and PE stages” section, as clearly shown in* ***Figures 5D-F*** *and in* ***Supplementary Figures 4E*** *and* ***F****.*

**8. This manuscript makes a lot of inferences, but lacks some reliable validation experiments.**

*Reply: We agree with the reviewer that some conclusions in our manuscript, particularly those regarding the activity of transcription factors, are based on predictions. However, we want to clarify that these predictions are founded on solid experimental evidence. They were indeed generated by integrating RNA-seq, ATAC-seq and ChIP-seq data, which also demonstrated significant mutual concordance.*

*As our primary goal of this work was to provide a reliable platform to study the development of the PE, we believe that our predictions offer a solid list of candidates for further experimental validation. In this study, we decided to validate the role of RARA in guiding PE specification, as Retinoic Acid represents the major driver of differentiation. We accomplished this by performing ChIP-seq on this receptor and combining it with ATAC-seq and RNA-seq upon RAR inhibition, thereby experimentally validating a part of the inferred network.*

*To better address the reviewer’s comment, we have now added a paragraph on the “Conclusions” section (lines 574-578). This paragraph acknowledges that our current findings are primarily descriptive and outlines our plans for future experimental work to explore the functional implications of the genomic elements identified in PE cells. We believe this addition adequately addresses their concerns and enriches the manuscript by clearly delineating the next steps for our research.*

Minor points: 
**1. Please provide the model and size of the labels font in the manuscript, as it is too cluttered.**

*Reply: Following the reviewer’s suggestion, we have adjusted font and text size.*

**2. There are still some language issues, please find the relevant mother tongue**

*Reply: We thank the reviewer for spotting the remaining language issues, that we have now corrected. Three of the authors who read the manuscript are native speakers, and the manuscript was finally evaluated and checked by the Stanford grant and research writing office of the Ob/Gyn department.*

**Second round of review**

**Reviewer 1**

The authors addressed all my previous concerns. I have no further comments.

**Reviewer 2**

The modifications were made quite carefully and met the publication standards.
